# Interface Stability and Kinetics of Sulfide Electrolytes in all‐Solid‐State Batteries

**DOI:** 10.1002/anie.202519663

**Published:** 2026-04-10

**Authors:** Kangli Wang, Wolfgang G. Zeier, Jürgen Janek, Doreen Mollenhauer

**Affiliations:** ^1^ Institute of Physical Chemistry Justus Liebig University Giessen Giessen Germany; ^2^ Center For Materials Research (LaMa) Justus Liebig University Giessen Giessen Germany; ^3^ Helmholtz Institute for Polymers in Energy Applications Jena (HIPOLE Jena) Jena Germany; ^4^ Helmholtz‐Zentrum Berlin für Materialien Und Energie GmbH (HZB) Berlin Germany; ^5^ Institute For Technical and Environmental Chemistry Friedrich Schiller University Jena Jena Germany; ^6^ Institute of Inorganic and Analytical Chemistry University of Münster Münster Germany; ^7^ Helmholtz Insitut Münster FZ Jülich Münster Germany

**Keywords:** chemical stability, DFT, electrochemical stability, interface, kinetics

## Abstract

All‐solid‐state batteries (ASSBs) are considered promising candidates for next‐generation energy storage systems, offering superior safety and energy density compared to conventional liquid‐based batteries. However, achieving stable long‐term cycling remains a significant challenge due to complex interfacial reactions, so interfacial stability has become a critical focus in the design and development of ASSBs. In this study, we present a comprehensive computational thermodynamic analysis of sulfide‐based solid electrolytes (SEs) and their various interfaces in ASSBs, with particular emphasis on cathode/SE, SE/interlayer, SE/coating, cathode/interlayer, cathode/coating and lithium‐silicon alloy/SE anode interfaces. The (electro)chemical stabilities of these interfaces are systematically evaluated. Our findings reveal that phosphate and sulfide‐type cathodes exhibit high thermodynamic stability when paired with sulfide SEs owing to favorable chemical bonding and compatibility. Furthermore, interlayers and coatings of cathode materials, such as phosphates and binary halides, notably improve interface stability by mitigating detrimental side reactions, making them particularly advantageous for long‐term cycling. For the lithium‐alloy anode, incorporation of silicon markedly improves stability by lowering the reaction energy, with the stabilization effect intensifying as the Si content increases. Kinetic analyses reveal that the interphase at Li*_x_* Si/Li_6_PS_5_Cl interface exhibits lower activation energy barriers for lithium‐ion migration compared to the bulk phases, thereby enhancing ionic transport.

## Graphical abstract

We reveal the chemical and electrochemical stability among cathode types and functional materials in contact with sulfide‐based electrolytes, the role of cathode charged states in interfacial reactions, and the corresponding volume changes at the interface. Furthermore, this work highlights enhanced stability and lithium‐ion transport at the a‐Li*_x_* Si/Li_6_PS_5_Cl interface, providing insights for optimizing ASSB performance.

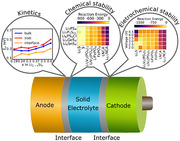

## 1 Introduction

Lithium‐ion batteries have been extensively employed for energy storage and power delivery, particularly in portable electronic devices and electric vehicles. As the demand for electrification continues to grow, the need for batteries with enhanced safety and higher energy densities has become increasingly critical. Substituting the liquid electrolyte in conventional lithium‐ion batteries with a SE can improve safety by reducing flammability, while also enhancing energy density, extending cycle life, and potentially enabling the use of alkali‐metal anodes [1, 2, 3]. Consequently, ASSBs have attracted significant attention as viable next‐generation battery systems. Although considerable progress has been made in ASSB technology, two key challenges remain the focus of intensive research: (i) the development of SEs with ionic conductivities comparable to or exceeding those of liquid electrolytes, and (ii) the formation of stable interfaces among the various ASSB components, including electrodes, SEs, and coating materials [4, 5, 6]. The first challenge has been successfully addressed over the past two decades with the development of SEs exhibiting high ionic conductivity, such as sulfide‐based electrolytes (up to 10^−2^ S/cm) [7, 8]. However, the second challenge, establishing stable interfaces, remains a significant barrier to the development of ASSBs.

To mitigate interface reactions, various methods have been proposed, including the insertion of an interlayer (such as buffer or protective layers) between the electrode material and the solid electrolyte, surface coatings, and the adoption of alloy anodes [9, 10, 11, 12, 13, 14, 15, 16, 17, 18, 19, 20, 21]. In addition to improving interfacial stability, these approaches have shown great potential for suppressing dendrite growth at the anode, accommodating volume changes, reducing interface resistances, and enhancing the effective ionic conductivity of composite electrodes, all of which collectively contribute to the improved performance and reliability of ASSBs. However, a limited understanding of the interface stability and kinetics of these interfaces remains. Furthermore, different interlayers, coating materials and lithium‐alloy anodes exhibit diverse (electro)chemical behaviors, highlighting the need for theoretical investigations of these interfaces.

In this study, we systematically investigated the chemical and electrochemical stability of various interfaces in common sulfide electrolytes tested in ASSBs through pseudo‐binary phase diagrams and grand potential phase diagrams, employing density functional theory (DFT) calculations [22, 23, 24, 25, 26, 27]. We identified the phase equilibria and decomposition reaction energies for cathode/SE, SE/interlayer, SE/coating, cathode/interlayer, cathode/coating, and Li*_x_* Si/SE interfaces. The interlayer materials considered in this study encompass buffer layers positioned between the cathode and the SE, and protective layers located between the anode material and the SE. Our results indicate that phosphate and sulfide‐based cathodes exhibit good (electro)chemical stability when paired with sulfide electrolytes. Additionally, a range of interlayer/coating materials, such as binary oxides, lithium multinary compounds, binary halides, phosphates, and binary sulfides, were systematically evaluated in contact with both the SE and the cathode, and their (electro)chemical stabilities were further compared. Finally, *ab initio* molecular dynamics (AIMD) simulations were employed to examine the formation and behavior of the Li*_x_* Si/Li_6_PS_5_Cl interface. The simulations reveal a lower activation energy for lithium‐ion migration within the interphase at the Li*_x_* Si/Li_6_PS_5_Cl interface than in the bulk phases, suggesting that the interphase enhances lithium‐ion transport and contributes to improved battery performance. Overall, our findings provide valuable insights into selecting optimal materials and interfaces in the construction of ASSBs using sulfide electrolytes.

## 2 Results and Discussion

### 2.1 Interfacial Stability

All studied materials, including sulfide electrolytes, cathode materials, interlayer/coating materials, and the lithium alloy anode, are summarized in Table 1. For the cathode materials, we consider both the fully discharged (lithiated) state and the fully or partially charged (delithiated) states of the selected materials. Regarding the partially charged state, the configuration stabilized at approximately 4 V is selected, as the safe operating voltage of lithium‐ion batteries typically ranges from slightly below 2 V to about 4 V. For alloy anodes, amorphous Li*_x_* Si (a‐Li*_x_* Si) is considered due to its high theoretical capacity and strong potential to prevent lithium metal nucleation and dendrite growth at low electrochemical potentials, enabling a higher energy density compared to other alloy anodes [28, 29, 30]. In this work, both chemical and electrochemical reaction energies are calculated to evaluate interfacial stability: the former evaluates whether two materials can chemically react to form more stable phases upon contact, while the latter assesses the interfacial stability under an applied voltage by incorporating the lithium chemical potential.

**TABLE 1:** List of materials studied in this work.

Category	Material Type	Compounds
Solid electrolytes	Sulfide	Li_3_PS_4_, Li_7_P_3_S_11_, Li_6_PS_5_Cl, Li_4_GeS_4_, Li_4_SnS_4_, Li_10_SnP_2_S_12_, Li_10_GeP_2_S_12_
Cathode materials (positive electrode)	Sulfide, sulfur	Li_2_S, S_8_
	Phosphates	Li_3_V_2_(PO_4_)_3_, LiV_2_(PO_4_)_3_, LiFePO_4_, FePO_4_, LiMnPO_4_, MnPO_4_
	Spinel‐type oxides	LiNi_0.5_Mn_1.5_O_4_ (LNMO), Ni_0.5_Mn_1.5_O_4_ (NMO), LiMn_2_O_4_, MnO_2_
	Layered oxides	LiNi_0.8_Co_0.15_Al_0.05_O_2_ (LNCA), Li_0.5_Ni_0.8_Co_0.15_Al_0.05_O_2_ (L_0.5_NCA), LiNi_0.33_Mn_0.33_Co_0.33_O_2_ (LNMC), Li_0.67_Ni_0.33_Mn_0.33_Co_0.33_O_2_ (L_0.66_NMC), LiNiO_2_, NiO_2_, LiCoO_2_, Li_0.5_CoO_2_
Interlayer/coating materials	Binary oxides	Y_2_O_3_, CuO, RuO_2_, SnO_2_, HfO_2_, ZnO, Fe_2_O_3_, CeO_2_, MgO, ZrO_2_, TiO_2_, Al_2_O_3_, SiO_2_, Li_2_O
	Lithium multinary oxides	Li_2_MoO_4_, Li_2_SiO_3_, Li_2_TiO_3_, Li_2_WO_4_, Li_2_ZrO_3_, Li_3_B_11_O18, Li_3_BO_3_, Li_3_NbO_4_, Li_3_VO_4_, LiB(CO_2_)_4_, LiTaO_3_, Li_4_Ti_5_O_12_, LiAlO_2_, LiNbO_3_, Li_2_PNO_2_, LiNO_3_, Li_4_SiO_4_
	Binary halides	BaF_2_, ZrF_4_, ZnF_2_, FeF_3_, YF_3_, PrF_3_, CaF_2_, CeF_4_, NiF_2_, SmF_3_, LaF_3_, MgF_2_, AlF_3_, LiF, LiCl, LiBr, LiI
	Phosphates	Co_3_(PO_4_)_2_, FePO_4_, Ni_3_(PO_4_)_2_, LaPO_4_, YPO_4_, AlPO_4_, Mn_3_(PO_4_)_2_, TiPO_4_, Li_3.5_Si_0.5_P_0.5_O_4_ (LSPO), Li_1.4_Al_0.4_Ti_1.6_(PO_4_)_3_ (LATP), Li_3_PO_4_, LiH_2_PO_4_
	Binary sulfides	ZnS, ZrS_2_, SiS_2_, TiS_2_, Sc_2_S_3_, Li_3_BS_3_, CdS, La_2_S_3_, HfS_2_, Li_3_PS_4_, Li_2_S, Li_2_Se
	Other	TiN, AlN, Li_2_CO_3_
Anode materials (negative electrode)	a‐Li*_x_* Si	a‐Li_22_Si_5_ (a‐Li_4.40_Si), a‐Li_13_Si_4_ (a‐Li_3.25_Si), a‐Li_7_Si_3_ (a‐Li_2.33_Si), a‐Li_12_Si_7_ (a‐Li_1.71_Si), a‐LiSi (a‐Li_1.00_Si), a‐LiSi_2_ (a‐Li_0.50_Si)

### 2.2 Cathode/SE Interfaces

**FIGURE 1:**
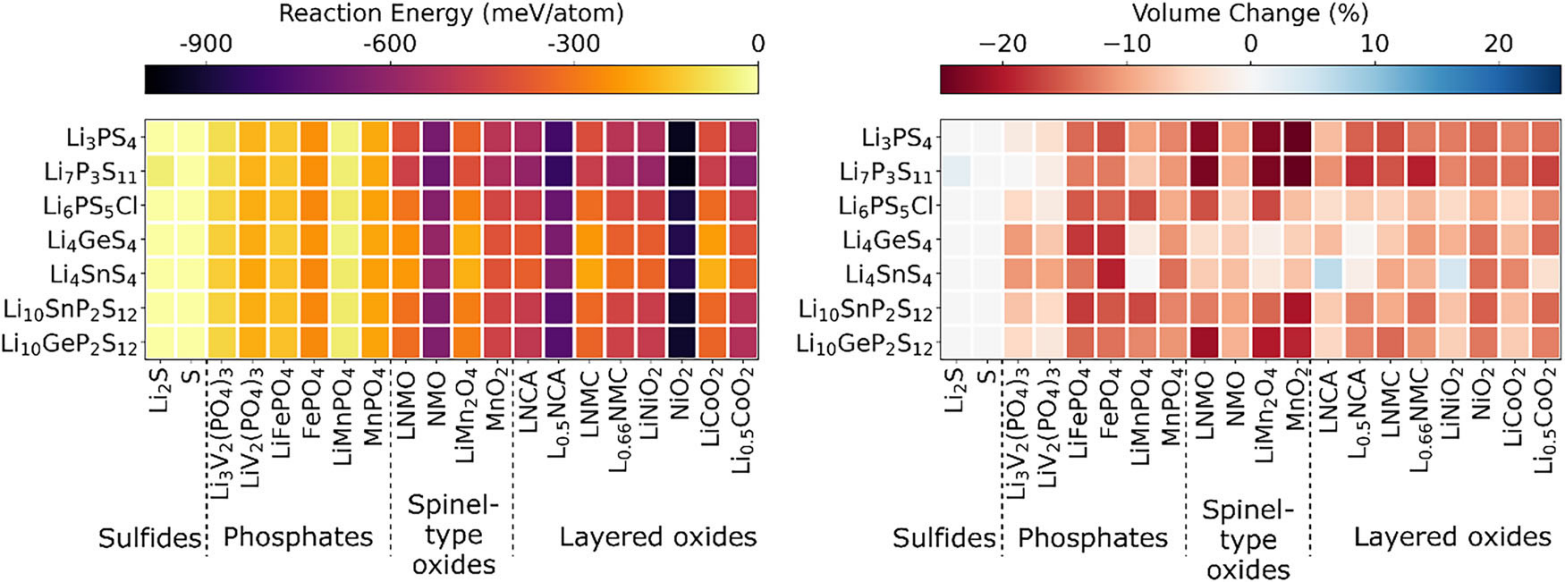
Chemical reaction energies (left) and percentage volume changes (right) associated with reactions at various cathode/SE interfaces calculated using density functional theory (DFT) with GGA density functional and GGA+U approach.

**FIGURE 2:**
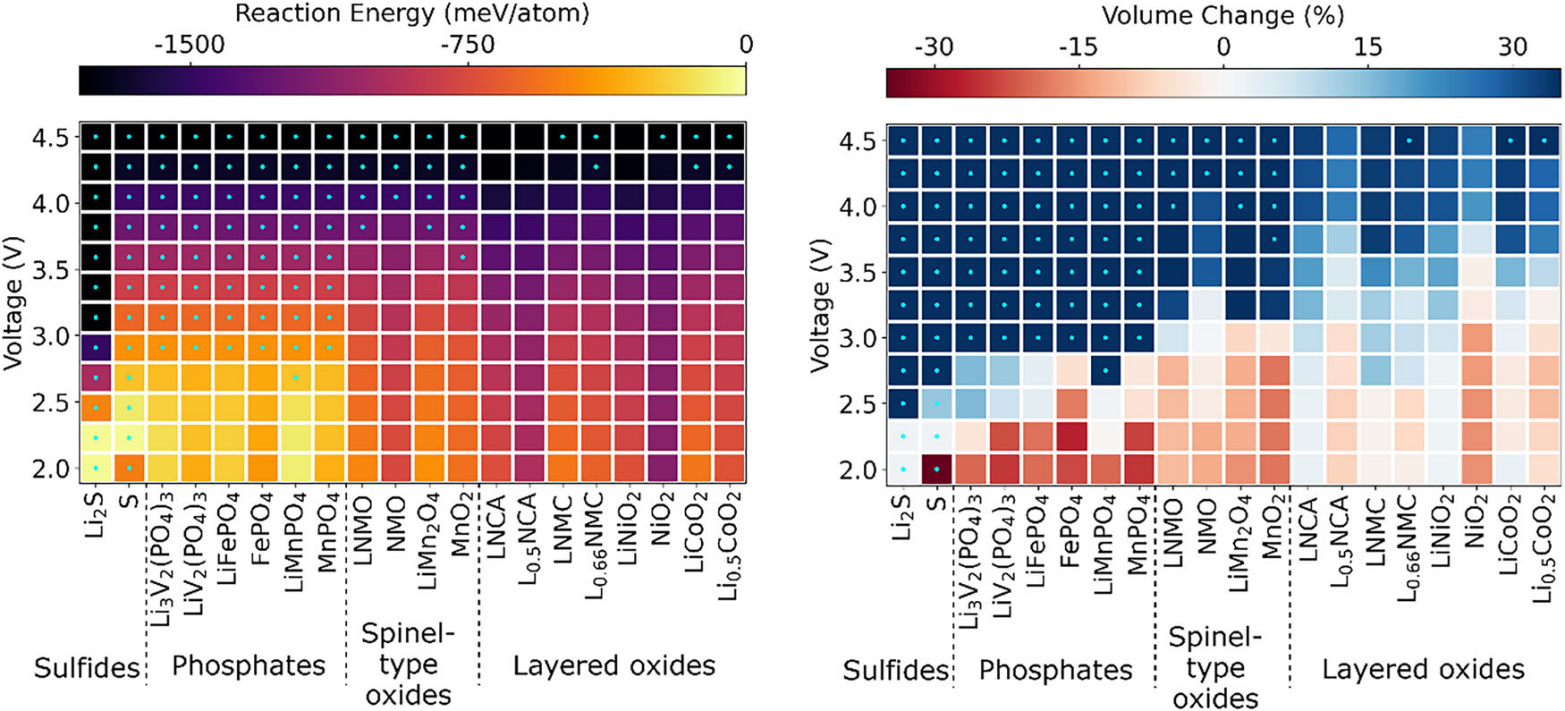
Electrochemical reaction energies (left) and corresponding volume changes (right) associated with reactions at various cathode/Li_6_PS_5_Cl interfaces calculated using DFT with GGA density functional and GGA+U approach. The dot represents the decomposition of the material at the interface, indicating the material decomposes with a more negative reaction energy than that of the interfacial reaction. Reactions without a dot represent direct interfacial reactions between two materials.

The chemical reaction energy of the sulfide electrolytes with various cathode materials is presented in Figure 1; the electrochemical reaction energy of the Li_6_PS_5_Cl electrolyte against different cathodes is displayed in Figure 2; additional electrochemical reaction energies of other cathode/SE interfaces are provided in Figures S3–S8.

From Figure 1, it is evident that the sulfide and phosphate‐type cathode materials exhibit less negative reaction energies (from 0.00 to ‐261 meV/atom) with sulfide electrolytes compared to the oxide cathode materials (from ‐175 to ‐963 meV/atom). This suggests that phosphate‐type and sulfide cathode materials are thermodynamically more stable in contact with sulfide electrolytes, which is consistent with previous studies [31, 32, 33, 34, 35]. This is also observed in electrochemical reactions as indicated in the Figure 2 and Figures S3–S8. The investigated phosphate‐type cathode materials containing PO_4_^3−^ anions possess strong P‐O bonds. These chemically stable covalent bonds reduce the reactivity of the phosphate cathode with the sulfide electrolytes. Sulfide cathode materials share chemical similarities with the sulfide electrolytes, leading to better chemical compatibility. Therefore, the lack of significant differences in bonding types reduces the driving force for reactions at the interface. In contrast, for oxide cathode materials the large chemical potential difference between oxygen in the cathode and sulfur in the electrolyte promotes interfacial reactions, leading to the ion mutual diffusion at the interface and the formation of byproducts such as Li_2_S, transition metal sulfides and/or transition metal oxides. The thermal instability of oxide cathode materials when combined with sulfide solid electrolytes is also observed experimentally [36].

Taking a closer look at oxide cathodes, layered oxide cathodes generally exhibit more negative (electro)chemical reaction energies with sulfide electrolytes compared to spinel oxide cathodes, indicating that interfaces formed by layered oxide cathodes are less stable when in contact with sulfide electrolytes. This observation agrees well with experimental work, which demonstrates that the use of a spinel phase rather than a layered oxide cathode can improve the cycling stability in ASSBs [37, 38]. Experimental studies have also demonstrated significant interfacial degradation between layered oxides such as LNCA and Li_6_PS_5_Cl during cycling [39]. Layered oxides consist of alternating layers of lithium and transition metals, with the transition metals typically occupying octahedral coordination sites; whereas spinel oxides have a more robust three‐dimensional (3D) framework, with lithium occupying tetrahedral sites and transition metals occupying octahedral sites. The structural differences between layered and spinel oxides can influence their interfacial reactivity with sulfide electrolytes, potentially contributing to the observed differences in chemical and electrochemical stability.

It is important to note that when sulfide SEs are in contact with phosphate‐type or certain oxide cathode materials (such as LiMn_2_O_4_ and MnO_2_), the decomposition of materials exhibits more negative reaction energy than the interface reactions at high voltages (indicated by dots in Figure 2). For example, at 3.75 V, the decomposition of Li_6_PS_5_Cl (into Li, P_2_S_7_, SCl and S_8_) has lower reaction energy than its interfacial reaction with MnO_2_. In contrast, the opposite trend is observed at lower voltages, where interfacial reactions become more favorable. This indicates that both material decomposition and interfacial reactions can occur with the dominant degradation pathway determined by which process is thermodynamically more favorable. The decomposition of materials is closely related to the limited electrochemical stability window of the materials, as illustrated in Figure S2. However, different behaviors are observed at other cathode/SE interfaces studied. For instance, at the Li_2_S/Li_6_PS_5_Cl interface, only the decomposition of Li_2_S and Li_6_PS_5_Cl, rather than any distinct interfacial reaction, is observed across the voltage range of 2.0∼4.5 V (Figure 2). Consequently, in this case, the decomposition of materials governs degradation processes across the entire considered voltage range. In contrast, for certain oxide cathode materials, such as LNCA and L_0.5_NCA, interfacial reactions dominate the degradation processes across the entire voltage range when interfaced with sulfide SEs, such as Li_6_PS_5_Cl, as shown in Figure 2. Overall, these results highlight the complex interplay between material stability, reaction pathways, and voltage‐dependent nature of degradation mechanisms.

In our calculations, the voltage‐dependent electrochemical reaction energies (referenced to Li/Li^+^) are generally more negative than the corresponding chemical reaction energies at fixed composition. This reflects the additional thermodynamic driving force introduced by the applied potential and is consistent with previous studies [40, 41]. In addition, both chemical and electrochemical reactions reveal that the charged (delithiated) state of considered cathodes exhibits more negative reaction energies with sulfide electrolytes compared to the discharged (lithiated) state. This is mainly attributed to the higher oxidation state of the transition metals in the charged state. In addition, the removal of lithium increases the number of vacancies in the charged state of cathode material, destabilizing the structure and further enhancing its reactivity with the sulfide electrolyte. Overall, this indicates that electrochemical reactions, especially when the cathode is in the charged state, play a key role in driving interfacial reactions.

In addition to reaction energy, the percentage volume changes (Δ*V*/*V*) for (electro)chemical reactions are estimated using the DFT‐relaxed volumes of reactants and products, weighted according to the ratios in the balanced reaction equation. A positive Δ*V*/*V* indicates an overall increase in volume during the reaction, which could lead to stress accumulation and potential cracking in the active material at the interface. Conversely, a negative Δ*V*/*V* reflects a reduction in volume, which may result in the formation of voids at the interface [40]. Most chemical reactions at the interface show volume reduction. This phenomenon can be attributed to the formation of stable, more compact, and denser products, such as Li_2_S and different transition metal sulfides (e.g., FeS_2_, MnS_2_). Concerning the electrochemical reactions in the voltage range of 2.0 to 4.5 V, our results indicate that at high voltage, the volume at the interface tends to increase significantly, whereas at low voltage, the volume either decreases or experiences only a slight increase. This behavior can be explained by several factors: (i) At high voltage, corresponding to the charged state, lithium atoms are extracted from the interface, leading to structural rearrangement. This rearrangement is accompanied by the formation of new phases with higher molar volumes. For instance, when NiO_2_ comes into contact with Li_6_PS_5_Cl at high voltage, the products, NiP_4_O_11_, SOCl_2_ and Ni(PO_3_)_2_, exhibit high molar volumes of approximately 131, 72 and 65 cm^3^/mol, respectively. (ii) At lower voltages, corresponding to the discharged state, the interface tends to incorporate lithium atoms, or the extent of lithium atom extraction is significantly reduced. During this process, the interface primarily forms denser products, resulting in volume reduction. For example, at low voltage, the interfacial reaction products at the LiNiO_2_/Li_6_PS_5_Cl interface include Ni_3_S_2_, Li_2_SO_4_, Li_3_PO_4_ and LiCl, with molar volumes of 40, 52, 48, and 21 cm^3^/mol, respectively. (iii) The formation of gaseous products, such as SO_2_ and O_2_, at the interface can also contribute to the observed volume reduction. In this study, the volume of gases is treated as zero, assuming that they leave the reaction zone.

### 2.3 SE/Interlayer and SE/Coating Interfaces

**FIGURE 3:**
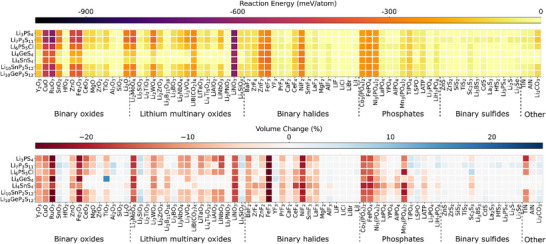
Chemical reaction energy (top) and percentage volume change (bottom) associated with reactions at various SE/interlayer and SE/coating interfaces calculated using DFT with GGA density functional and GGA+U approach.

The chemical and electrochemical reaction energies of various SE and interlayer or coating interfaces are illustrated in Figures 3 and Figures S9–S15, respectively. Compared to the reaction energies observed at the cathode/SE interfaces, the incorporation of interlayer or coating materials significantly enhances interfacial stability and mitigates side reactions. Among all the interlayer/coating materials investigated, binary sulfide materials exhibit the highest interfacial stability with sulfide electrolytes due to their similar chemical environment. This is consistent with previous theoretical and experimental findings on the high interfacial stability of sulfide materials with sulfide electrolytes [40, 42, 43, 44, 45]. Phosphate and binary halide materials also show strong interfacial stability due to the strong P‐O covalent bonds in phosphate structures and the ionic bonds found in metal halides. Binary oxides and lithium multinary oxides present a broader range of chemical reaction energies with sulfide electrolytes, spanning from 0 to ‐677 meV/atom. While some oxides, such as Al_2_O_3_, SiO_2_ and HfO_2_ exhibit strong stability with sulfide electrolytes, other oxides like CuO and RuO_2_ are more reactive. This indicates that the effectiveness of oxide interlayer/coating materials is highly dependent on the specific material.

Regarding the electrochemical stability (Figure S9–S15), we find that at low voltages, the electrochemical reaction energies at the SE/interlayer or SE/coating interfaces are minimal. As the voltage increases, the electrochemical reaction energies rise, suggesting a voltage‐dependent instability at these interfaces. Similar to the cathode/SE interfaces discussed earlier, three degradation mechanisms are identified at SE/interlayer or SE/coating interfaces in the voltage range of 2∼4.5 V: (i) degradation governed entirely by interfacial reactions, (ii) degradation dominated by the decomposition of materials, and (iii) a combination of interfacial reactions and decomposition. These processes are accompanied by voltage‐dependent volumetric changes at the interface, with significant expansion at high voltages and either slight expansion or negative volume changes at low voltages.

### 2.4 Cathode/Interlayer and Cathode/Coating Interface

**FIGURE 4:**
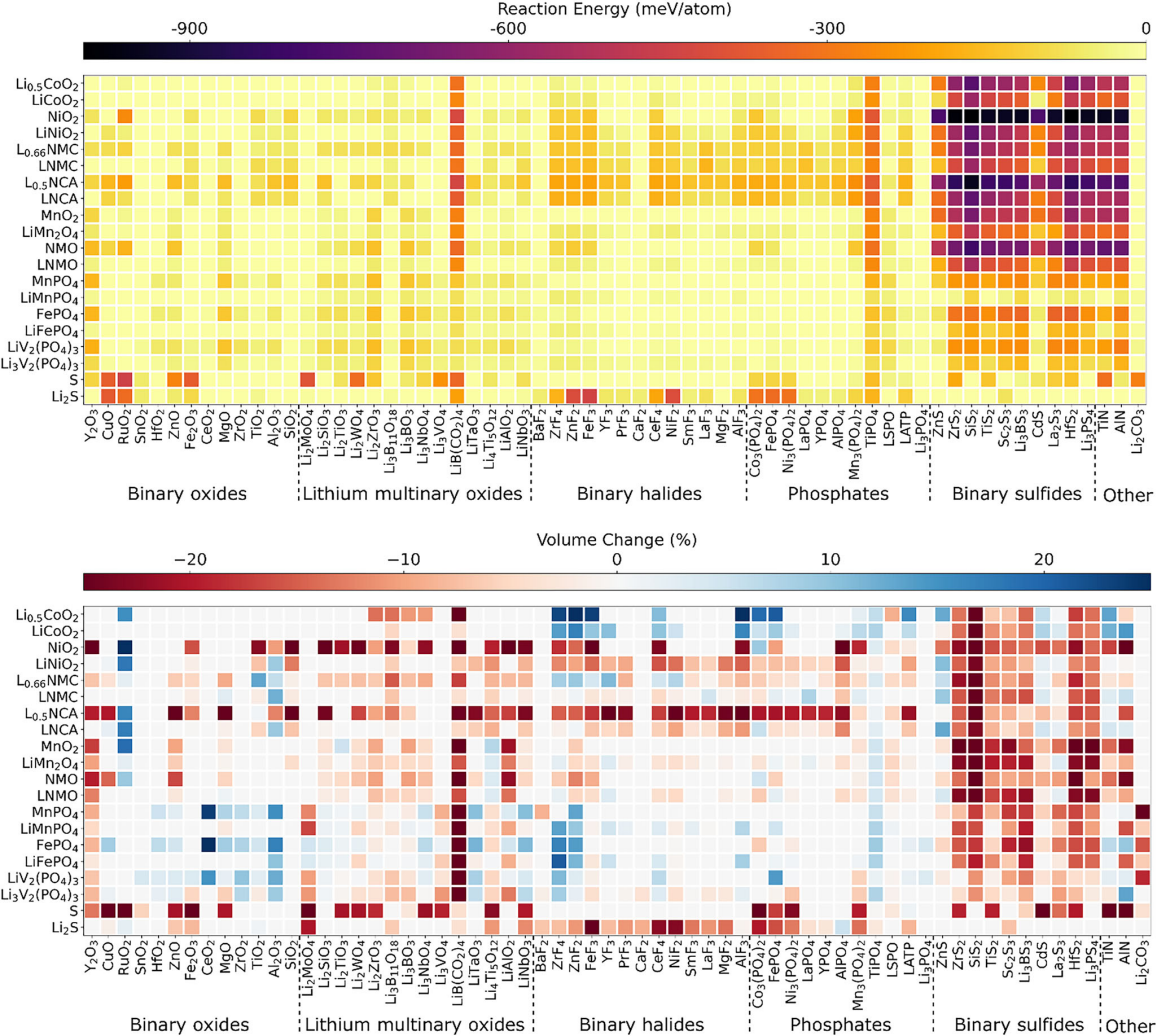
Chemical reaction energy (top) and percentage volume change (bottom) associated with reactions at various cathode/functional material interfaces calculated using DFT with GGA density functional and GGA+U approach.

The calculated chemical reaction energies of various cathode/interlayer and cathode/coating interfaces are illustrated in Figure 4. Here, we only consider buffer layer and coating materials employed at the cathode. Our results reveal that binary oxide, lithium multinary oxide, binary halide, and phosphate materials exhibit only small negative reaction energies when paired with the cathodes considered. This suggests that these materials can effectively prevent interfacial reactions. The stability of the binary oxide in contact with cathode material is attributed to either the chemical compatibility between oxide interlayers/coatings and oxide cathodes or the inherent stability of phosphate and oxide cathodes. The high interfacial stability observed in lithium multinary oxide, binary halide, and phosphate materials is primarily driven by the presence of strong chemical bonds: metal‐oxygen covalent bonds in lithium multinary oxides, ionic bonds in binary halide oxides, and phosphorus‐oxygen bonds in phosphate materials. In contrast, materials consisting of sulfides and nitrides exhibit higher reactivity when contact with cathode material, particularly at the interfaces with oxide cathodes. This behavior differs from that observed at SE/interlayer or SE/coating interfaces, suggesting that the selection of interlayer or coating materials must take into account not only their stability with the SE but also their compatibility with the cathode material.

### 2.5 a‐Li*_x_* Si/SE interfaces

**FIGURE 5:**
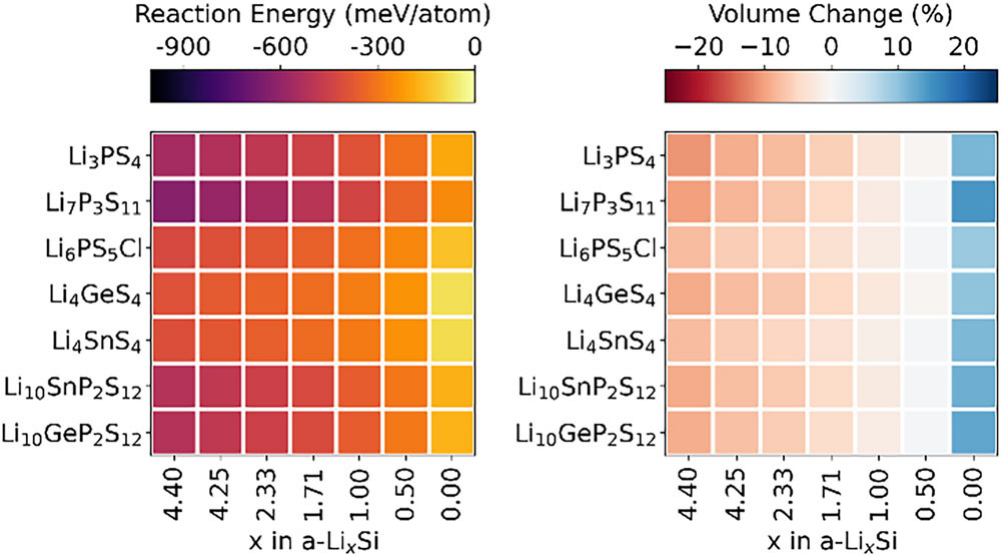
Chemical reaction energy (left) and percentage volume change (right) associated with reactions at various a‐Li*_x_* Si/SE interfaces.

As displayed in Figure 5, the chemical reaction energy at the interface of a‐Li*_x_* Si with sulfide SE becomes less negative as *x* increases, indicating an enhancement in interfacial stability with the incorporation of Si. This improvement becomes more pronounced as the silicon content increases. Moreover, at high *x* values, the interface experiences a negative volume change, which gradually decreases as *x* decreases, reaching a minimum within the range of *x* = 1.00 ∼ 0.50. Beyond this range, the interface exhibits expansive behavior. This volume change is mainly attributed to the formation of SiS_2_ at the low *x* values, which shows a more expansive structure. In terms of electrochemical stability (Figures S16–S22), the interface stability was evaluated across a voltage range of 0 to 2.5 V due to the low electrochemical stability window of Li*_x_* Si alloy. Here, the interfacial reaction energies are substantially negative.

### 2.6 Kinetic Properties

In addition to the computational thermodynamic analysis, we further investigated the interfacial kinetics through AIMD simulations to capture the dynamic behavior and atomic‐scale evolution at the interface. Given the enhanced interfacial stability achieved by Si incorporation into lithium anodes, we selected a‐Li*_x_* Si and crystalline Li_6_PS_5_Cl, along with their interface, as our model system for in‐depth studies of interfacial kinetics.

### 2.7 Bulk and Slab

**FIGURE 6:**
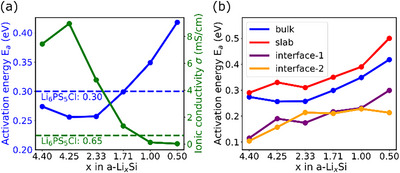
(a) Activation energy and corresponding ionic conductivity of bulk a‐Li_x_Si and Li_6_PS_5_Cl at room temperature; (b) activation energy of bulk a‐Li_x_Si, slab a‐Li_x_Si, and the a‐Li_x_Si/Li_6_PS_5_Cl. Interface‐1 and interface‐2 correspond to slab‐1 and slab‐2 of Li_6_PS_5_Cl, respectively, as shown in Figure 7.

The Arrhenius plots for all considered systems are presented in Figures S23–S25. The calculated activation energy and ionic conductivity of a‐Li*_x_* Si and Li_6_PS_5_Cl, along with the activation energy of slab a‐Li*_x_* Si and the a‐Li*_x_* Si/Li_6_PS_5_Cl interface, are presented in Figure 6. For Li_6_PS_5_Cl, the activation energy of ionic jumps is 0.30 eV, with an ionic conductivity at room temperature of 0.65 mS/cm, aligning well with experimental results [46, 47, 48]. For a‐Li*_x_* Si, the calculated activation energy shows an initial decrease with decreasing *x*, followed by an increase. The ionic conductivity shows the opposite trend. When *x* = 4.25, the lowest activation energy and the highest ionic conductivity are found. These observations are consistent with the findings from previous studies [49]. The incorporation of a small amount of Si introduces structural heterogeneity, which provides additional space and diffusion pathways, facilitating lithium ion migration. As *x* decreases, the fraction of lithium atoms decreases, leading to the formation of Si clusters (or a more interconnected Si network) [50, 51]. This structural evolution reduces the availability of continuous lithium diffusion pathways, thereby hindering lithium atom transport. The evolution of the average coordination number of Si‐Si pairs over simulation time (Figure S26) shows a clear increase with lower values of *x*, further supporting our above conclusion.

**FIGURE 7:**
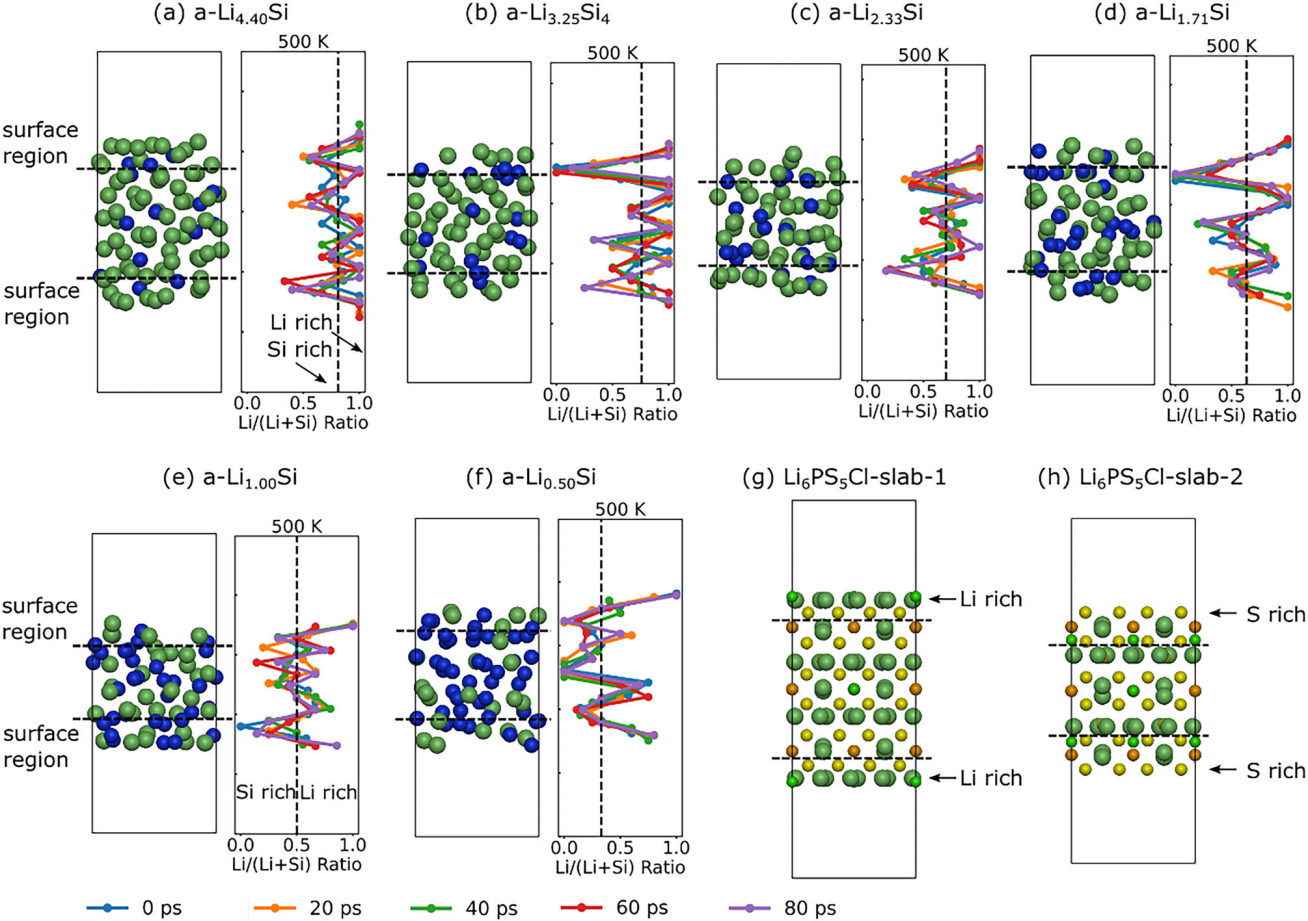
Atomic configurations of slab a‐Li*_x_* Si and Li_6_PS_5_Cl with the Li/(Li+Si) ratio plotted along the z‐axis of slab a‐Li*_x_* Si over the simulation time. Li, Si, P, S and Cl atoms are represented by dark green, blue, orange, yellow and light green balls, respectively. Solid lines in different colors in the right panel indicate different simulation times.

For a given composition, the surface activation energy for lithium‐ion migration across the surface of an a‐Li*_x_* Si slab is comparable to or greater than that of its bulk counterpart (Figure 6b). Structurally, atoms in the surface region (defined in this work as the outermost 2 Å along the z‐axis) undergo rearrangement due to the presence of a vacuum. As shown in Figure 7, the Li/(Li+Si) ratio indicates that lithium atoms tend to concentrate at the outer surface, while silicon atoms are more likely to remain beneath the lithium layer. This accumulation of lithium at the outer surface and the positioning of silicon beneath can hinder lithium‐ion mobility in the surface region, thereby creating a barrier to ion migration. A similar lithium‐rich outer surface was also observed in prior work on vacuum/Li*_x_* Si/Cu interfaces [52]. For crystalline Li_6_PS_5_Cl, two (001) surfaces are considered in this work: lithium‐rich and sulfur‐rich surfaces. The lithium‐rich surface (slab‐1) exhibits lower activation energies (0.23 eV) for ionic diffusion than the bulk due to under‐coordinated surface atoms. The reduced coordination of surface lithium atoms facilitates more frequent hopping between available sites. When lithium atoms are positioned beneath an S layer (slab‐2), they are less exposed to the surface and experience more restricted diffusion pathways compared to slab‐1. The sulfur atoms slightly hinder lithium mobility, resulting in a higher activation energy (0.28 eV) than the lithium‐rich surface but still lower than that of the bulk.

### 2.8 a‐Li*_x_* Si/Li_6_PS_5_Cl interfaces

**FIGURE 8:**
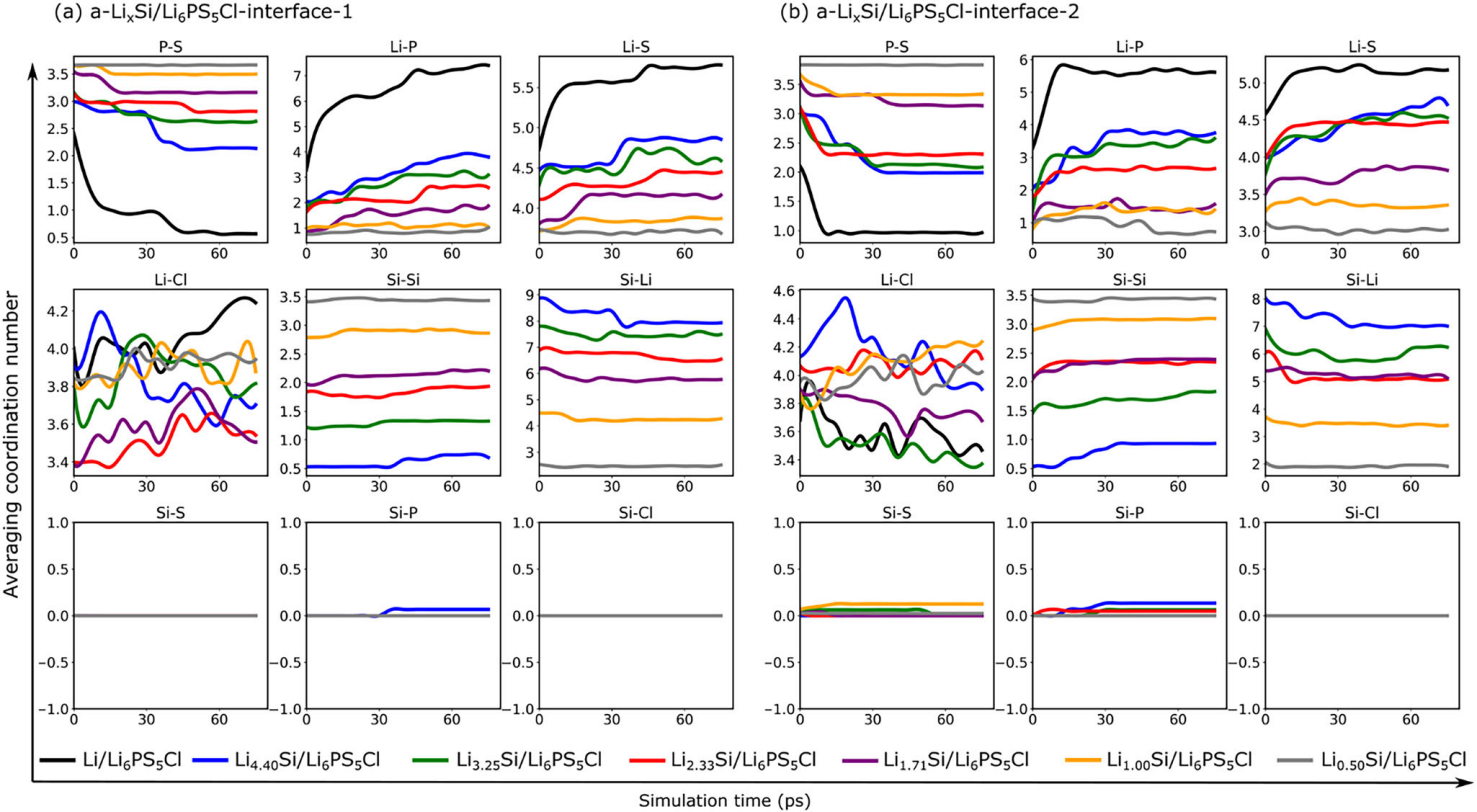
Evolution of the average coordination number for the a‐Li_x_Si/Li_6_PS_5_Cl‐1 and a‐Li_x_Si/Li_6_PS_5_Cl‐2 interface models at 298 K. The averaging is carried out over elements and over time, with a time step of 5 ps.

Figure 8 illustrates the evolution of the average coordination number at 298 K between the selected pairs of atoms in the a‐Li*_x_* Si/Li_6_PS_5_Cl‐1 and a‐Li*_x_* Si/Li_6_PS_5_Cl‐2 interface structures. These structures correspond to the slab‐1 and slab‐2 configurations of Li_6_PS_5_Cl, respectively. For the Li/Li_6_PS_5_Cl, a continuous decrease in the number of P‐S bonds is observed, especially during the first 15 ps, whereas the number of Li‐S and Li‐P contacts increases. This indicates the decomposition of the PS_4_^3−^ group and the formation of new Li‐S and Li‐P bonds, suggesting significant chemical reactivity. Additionally, the number of Li‐Cl interactions oscillates throughout the simulation period. These observations align well with previous computational and experimental studies on the Li/Li_6_PS_5_Cl interface [53, 54, 55, 56]. Upon introducing Si into the lithium anode, there is a clear reduction in the decomposition of the PS_4_^3−^ group. This reduction becomes more pronounced as the *x* deceases. Furthermore, the formation of Li‐S and Li‐P contacts is substantially diminished with decreasing *x*. These observations indicate that the incorporation of Si can enhance the interfacial stability, which agrees well with experimental studies on Li*_x_* Si/Li_6_PS_5_Cl [49, 57, 58, 59]. Moreover, increasing Si content within the lithium anode improves this stability further. This aligns with our previous analysis on the interfacial stability. Additionally, we observe an increase in Si‐Si bonds and a decrease in Li‐Si bonds over time, with minimal formation of Si‐S, Si‐P, and Si‐Cl bonds. As *x* decreases, the number of Si‐Si bonds grows significantly, accompanied by a reduction in the number of Li‐Si bonds. Despite the reduction in Li‐Si bond counts, the average Li‐Si coordination number remains high (ranging from 4 to 9). These strong Si‐Si and Li‐Si bonds contribute to a more stable interfacial environment, effectively mitigating degradation processes.

For a given composition, the interphase at a‐Li*_x_* Si/Li_6_PS_5_Cl interface exhibits a lower activation energy for the lithium ion migration compared to the bulk phases, with values ranging from 0.1 to 0.3 eV. This indicates that the interphase provides more efficient pathways for lithium transport. Notably, the activation energy at the a‐Li*_x_* Si/Li_6_PS_5_Cl interface generally tends to increase as *x* decreases, with the lowest activation energy observed for the Li_4.40_Si/Li_6_PS_5_Cl interface. We observe similar phenomena for both the a‐Li*_x_* Si/Li_6_PS_5_Cl‐1 and a‐Li*_x_* Si/Li_6_PS_5_Cl‐2 structures, indicating consistent interfacial behavior across the different slab configurations. Our previous analysis of the a‐Li*_x_* Si slab suggests that lithium atoms tend to concentrate at the outer surface, effectively creating a reservoir of mobile lithium ions that can more readily participate in ionic transport across the interface. Additionally, in crystalline Li_6_PS_5_Cl, the 48 h sites are only partially occupied by lithium ions, offering readily accessible hopping sites that facilitate lithium ion movement. The combination of a structurally disordered a‐Li*_x_* Si layer adjacent to crystalline Li_6_PS_5_Cl introduces a mismatch in atomic arrangements, potentially creating favorable pathways for lithium migration at the interface. These factors contribute to the lower activation energy found in the interphase at the a‐Li*_x_* Si/Li_6_PS_5_Cl interface. This conclusion is well supported by a recent theoretical study employing machine learning force field (MLFF)‐based molecular dynamics (MLMD) simulations [60]. The study found that the formed interphase at the Li*_x_* Si/Li_6_PS_5_Cl interface exhibits an exceptional ionic conductivity of 27.2 mS/cm, a value significantly higher than that of either bulk Li*_x_* Si or Li_6_PS_5_Cl. Moreover, an experimental study on the natural interlayer between lithium metal and Li_6_PS_5_Cl showed lower conductivity of the interphase [61]. These results suggest that the specific interphase formed at the Li*_x_* Si/Li_6_PS_5_Cl interface is critical for facilitating ionic transport [49].

## 3 Conclusions

In this work, we present a comprehensive analysis of interfacial stability and kinetics of sulfide‐based electrolytes in ASSBs. In general, phosphate and sulfide cathodes are thermodynamically more stable in contact with sulfide electrolytes compared to oxide cathodes. Among the oxide cathodes, spinel structures exhibit higher stability than layered structures. Our analysis also reveals that electrochemical reactions at the electrolyte/cathode interface exhibit higher reaction energies than chemical reactions, with the charged state of cathodes showing more negative reaction energies than the discharged state. This indicates that electrochemical interactions, especially with charged cathodes, predominantly drive interfacial reactions during battery cycling. Concerning interlayer/coating materials, binary sulfide, phosphate and binary halide materials show strong interfacial stability with sulfide electrolytes; while binary oxide, lithium multinary oxide, binary halide and phosphate materials exhibit only marginally negative reaction energies with the considered cathode materials. Three degradation mechanisms are identified from the electrochemical stability analysis at sulfide SE related interfaces within the voltage range of 2∼4.5 V: (i) degradation governed entirely by interfacial reactions, (ii) degradation dominated by the self‐decomposition of materials, and (iii) a combination of interfacial reactions and self‐decomposition. In addition, both thermodynamic and kinetic analyses indicate that the incorporation of Si into the lithium anode significantly improves interfacial stability. Increasing the Si content in the anode further enhances stability. Notably, the interphase at the a‐Li*_x_* Si/Li_6_PS_5_Cl interface shows a lower activation energy for lithium‐ion migration than bulk phases, suggesting its potential to enhance lithium‐ion transport and improve overall battery performance. Overall, this work offers a deep understanding of the interfacial mechanisms in ASSBs, providing valuable guidance for material selection and design strategies to enhance stability and enable long‐term cycling performance.

## Conflicts of Interest

The authors declare no conflicts of interest.

## Supporting information



The authors have cited additional references within the Supporting Information [62, 63, 64, 65, 66, 67, 68, 69, 70, 71, 72, 73, 74, 75, 76, 77, 78].**Supporting File 1**: anie71918‐sup‐0001‐SuppMat.pdf.

## Data Availability

The data that support the findings of this study are available in the Supporting Information of this article.
