# Supplementary material for: Interface Stability and Kinetics of Sulfide Electrolytes in all‐Solid‐State Batteries
Source: Angew Chem Int Ed Engl. 2026 Apr 10;65(21):e19663. doi: 10.1002/anie.202519663 (PMC13182192; doi:10.1002/anie.202519663)
Supplement: Supplementary file 1 — The authors have cited additional references within the Supporting Information [62, 63, 64, 65, 66, 67, 68, 69, 70, 71, 72, 73, 74, 75, 76, 77, 78].Supporting File 1: anie71918‐sup‐0001‐SuppMat.pdf. [file ANIE-65-e19663-s001.pdf]

Supporting Information (SI)

Interface Stability and Kinetics of Sulfide Electrolyte in All-Solid-State Batteries

Kangli Wang[1-5], Wolfgang G. Zeier[6,7], Jürgen Janek[1,2], and Doreen Mollenhauer[1-5,*]

[1] *Institute of Physical Chemistry, Justus-Liebig University Giessen, Heinrich-Buff-Ring 17, 35392 Giessen, Germany*

[2] *Center for Materials Research (LaMa), Justus-Liebig University Giessen, Heinrich-Buff-Ring 16, 35392 Giessen, Germany*

[3] *Helmholtz Institute for Polymers in Energy Applications Jena (HIPOLE Jena), Lessingstrasse 12-14, 07743 Jena, Germany*

[4] *Helmholtz-Zentrum Berlin für Materialien und Energie GmbH (HZB), Hahn-Meitner-Platz 1, 14109 Berlin, Germany*

[5] *Institute for Technical and Environmental Chemistry, Friedrich Schiller University Jena, Philosophenweg 7a, 07743 Jena, Germany*

[6] *Institute of Inorganic and Analytical Chemistry, University of Münster, Corrensstrasse 30, 48149 Münster, Germany*

[7] *Helmholtz Insitut Münster, FZ Jülich, Corensstrasse 46, 48149 Münster, Germany*

E-mail: doreen.mollenhauer@helmholtz-berlin.de; doreen.mollenhauer@uni-jena.de

1 Computational details

Materials selection. If available, the crystal structures and precomputed energies were obtained from the Materials Project (MP) database.[1] In cases where these were not available, initial crystal structures were derived from Inorganic Crystal Structure Database (ICSD) or experimental work.[2] Sources of considered compounds were collected in Table S1. For disordered structures, 100 potential atomic configurations were randomly generated, and the lowest-energy configuration was selected for further analysis.

DFT calculations. All DFT calculations in this study were performed using the Vienna Ab-initio Simulation Package (VASP) within the projector augmented-wave approach.[3, 4, 5, 6] The Perdew-Burke-Ernzerhof (PBE) generalized gradient approximation (GGA) was employed for all

calculations.[7, 8, 9] For thermodynamic analysis, the computational parameters, such as the plane-wave energy cutoff and k-point density, were selected to be consistent with MP. Additionally, energy correction schemes for elements were applied following the protocols established by the MP.[10, 11] For slab and interface structures, self-consistent field convergence was achieved when the energy change was less than 10^{-5} eV, and the convergence criterion for ionic relaxations was set to atomic forces below 0.01 eV/Å. The energy cutoff of the plane-wave basis expansion was set to 520 eV with a Gaussian smearing width of 0.05 eV.

Evaluation of interfacial stability. Chemical stability of an interface is assessed by calculating the reaction energy through the construction of a pseudobinary phase diagram.[12, 13, 14, 15, 16, 17, 18] The ratio corresponding to the most negative reaction energy is identified as the optimal composition. The interface composition is given by:

$$C_{interface}(c_A, c_B) = x \cdot c_A + (1 - x) \cdot c_B \tag{1}$$

where c_A and c_B represent the specific compositions of the interface, normalized to one atom per formula unit; x is the atomic fraction of c_A, which varies from 0 to 1. The chemical reaction energy $\Delta E(c_A, c_B)$ of the interface pseudo-binary is calculated by:

$$\Delta E(c_A, c_B) = \min_{x\in[0,1]} \frac{1}{N}[E_{eq,interface}(xc_A + (1 - x)c_B) - xE(c_A) - (1 - x)E(c_B)] \tag{2}$$

Here $E(c_A)$ and $E(c_B)$ are the DFT-calculated energies of the interface compositions c_A and c_B, respectively; $E_{eq,interface}$ is energy of phase equilibrium at a given composition; N is the number of atoms involved in the reaction, serving as the normalization factor. The percentage volume change is computed as:

$$\Delta V/V = \frac{V_{eq,interface}(xc_A + (1 - x)c_B) - xV(c_A) - (1 - x)V(c_B)}{xV(c_A) + (1 - x)V(c_B)} \times 100\% \tag{3}$$

where $V(c_A)$ and $V(c_B)$ are the DFT-relaxed volumes of compositions c_A and c_B, respectively; $V_{eq,interface}(xc_A + (1 - x)c_B)$ is the total volume of the reaction products at the equilibrium composition.

To evaluate the electrochemical stability of an interface, the grand potential phase diagram with respect to lithium was constructed.[12, 13, 14, 15, 16, 17, 18] The applied electrostatic potential ϕ was incorporated into the lithium chemical potential μ_{Li} as follows:

$$\mu_{Li}(\phi) = \mu^0_{Li} - e\phi \tag{4}$$

Where μ^0_{Li} is the chemical potential of lithium metal, and the potential ϕ is referenced to the lithium metal in this study. The electrochemical reaction energy at a given applied voltage ϕ is calculated via

$$\Delta E(c_A, c_B, \phi) = \min_{x\in[0,1]} \frac{1}{N_{gc}}[E^{\phi}_{eq}(xc_A + (1 - x)c_B) - xE^{\phi}(c_A) - (1 - x)E^{\phi}(c_B)] \tag{5}$$

where N_{gc} is the normalization factor, representing the total number of atoms excluding lithium. Both the pseudo-binary phase diagram and grand potential phase diagram were constructed using the Python Materials Genomics (Pymatgen) package.[19]

AIMD calculations. All AIMD calculations are performed using a plane wave basis set with an energy cutoff of 300 eV and 1×1×1 Monkhorst-Pack k-point sampling. For all structures, the lattice constants along three directions are more than 8 Å. For the generation of amorphous Li_xSi alloys, initial crystalline structures were used as starting configurations. In order to obtain the randomized structures, the models were first heated to 1500 K (above the melting point of alloy) for 15 ps with a time step of 2 fs, using constant volume and temperature (NVT) AIMD calculations controlled by a Nosé thermostat. The pair correlation function of each model were monitored to ensure its convergence. Afterward, the structures were rapidly quenched to 0 K at a cooling rate of 0.5 K/fs. The temperature during this process was controlled via velocity rescaling. Finally, the quenched structures were refined through careful volume optimization using first-principles total energy minimization calculations. The obtained energy and volume of amorphous alloy were used for further analysis.

For the generation of interface structure, we use Figure S1 to illustrate the construction process. Starting from the bulk alloy, the initial structure is taken from bulk lithium by substituting part of lithium with Si atoms as displayed in stage I. The lattice constants a and b of the amorphous Li_xSi alloy remain identical to those of the Li_6PS_5Cl electrolyte. The lattice constant c is determined using the relation $V/(a \times b)$, where V represents the volume of the amorphous alloy, as obtained from the preceding analysis. The bulk structure is heated to 1500 K for 15 ps and subsequently quenched to 0 K (Stage II and III), following the same procedures outlined above. The resulting structure is further optimized using DFT (Stage IV). During the volume optimization, the lattice constants a and b of the alloy are kept fixed, while only the lattice constant c is varied. For alloy slabs, the presence of vacuum and flat surfaces can influence atomic distribution. Therefore, we conduct several AIMD simulations in the canonical ensemble with varying temperatures. The initial slab model is constructed by introducing a 15-Å-thick vacuum gap in the z-direction within the bulk alloy (Stage V). The slab is annealed at 800 K for 12 ps and subsequently at 600 K for 12 ps, followed by relaxation at 300 K for 6 ps, allowing for adequate atomic redistribution and relaxation (stage VI). In the final step, this slab structure is relaxed through DFT calculation at 0 K (Stage VII). After obtaining the a-Li_xSi and Li_6PS_5Cl slabs, the interface is constructed and further optimized.

To investigate the lithium-ion transport properties, AIMD simulations were conducted using an NVT ensemble over 80 ps, with a time step of 1 fs, at various temperatures. The a-Li_xSi alloy bulk, slab, and interface were simulated at temperatures ranging from 500 K to 900 K, while the sulfide electrolyte was simulated between 500 K and 1200 K, all of which are below the respective melting points of the materials. The diffusivity (D) of lithium ions at each temperature was determined using the Einstein

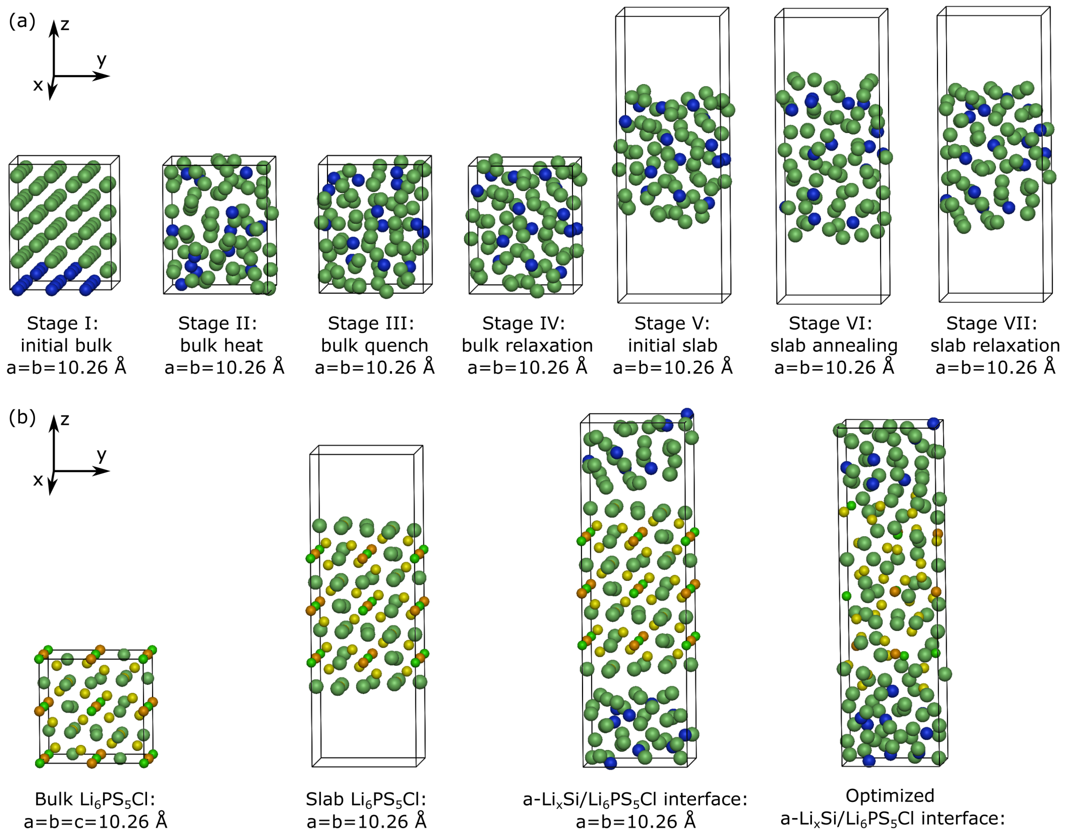

Figure S1: Atomic configurations for a-Li_xSi alloy and Li_6PS_5Cl: bulk, slab and interface at different simulation stages.

relation[20]

$$D = \frac{1}{2n} \frac{\mathrm{MSD}(\Delta t)}{\Delta t} \tag{6}$$

where n is the dimension of the system, $\mathrm{MSD}(\Delta t)$ is the mean squared displacement of lithium ions over the time interval Δt. To accurately assess the dynamics of lithium ions at the surface and interface, lithium atoms are included in the mean squared displacement (MSD) calculation if they remain within the defined surface or interface region for at least 50 ps out of 80 ps. The surface region and interface region are defined with a thickness of 2 and 5 Å, respectively, along z-direction. In the case of the Li_6PS_5Cl-slab-2, where some lithium atoms are found buried beneath the surface, the thickness of the surface region is extended to 3 Å, while the interface region with a-Li_xSi is extended to 6 Å.
The diffusivity coefficient in a solid generally follows an Arrhenius relationship[20]

$$D = D_0 \exp\left(-\frac{E_a}{kT}\right) \tag{7}$$

where D_0 is the pre-exponential factor, E_a is the activation energy, k is the Boltzmann constant, and T is the temperature. To obtain room temperature diffusivity coefficient and activation energy, an Arrhenius plot of the log of the dffusivity coefficient versus $1/T$ is constructed. The Li^+ ionic conductivity at room temperature of 300 K can then be derived from the Nernst-Einstein equation as follows[20]

$$\sigma_{Li^+} = \frac{Nq^2}{VkT} D \tag{8}$$

where N, q, and V are the number of ions, the charge of the mobile-ion species, and the volume of the system, respectively.

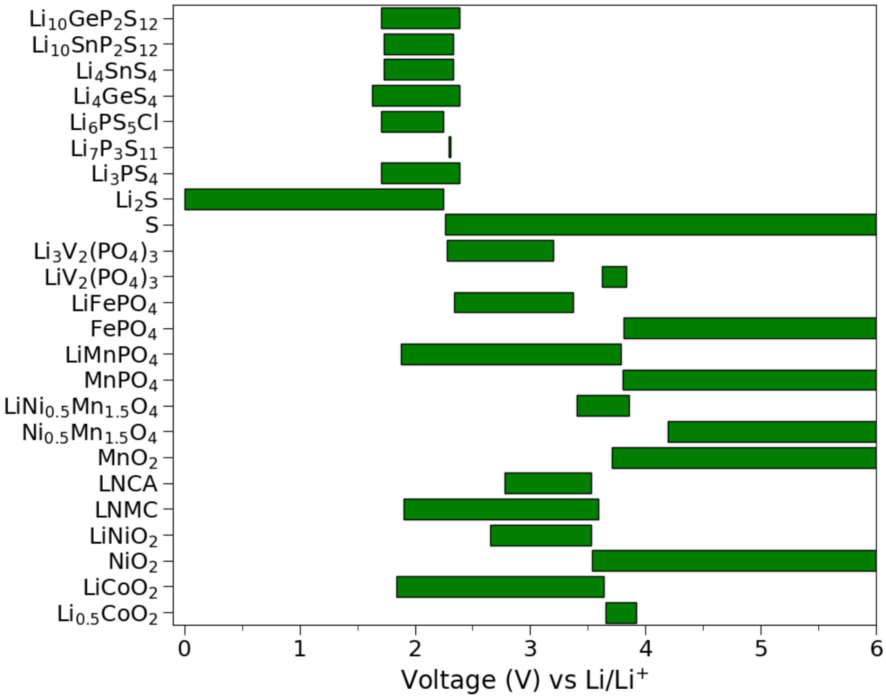

Figure S2: Electrochemical stability window of compounds calculated using DFT with the GGA density functional and GGA+U approach.

Compound	Source	Compound	Source
Li_3PS_4	ICSD-180318	$Li_7P_3S_{11}$	mp-641703
Li_6PS_5Cl	ICSD-259205	Li_4GeS_4	mp-30249
Li_4SnS_4	mp-1195718	$Li_{10}SnP_2S_{12}$	mp-721236
$Li_{10}GeP_2S_{12}$	mp-696138	Li_2S	mp-1153
S_8	mp-96	$Li_3V_2(PO_4)_3$	ICSD-96962
$LiV_2(PO_4)_3$	ICSD-96964	$LiMnPO_4$	mp-18997
$MnPO_4$	mp-26980	$LiFePO_4$	mp-19017
$FePO_4$	mp-20361	$LiNi_{0.5}Mn_{1.5}O_4$	ICSD-94762
$LiMn_2O_4$	ICSD-40485	MnO_2	mp-25275
$LiNi_{0.8}Co_{0.15}Al_{0.05}O_2$	ICSD-257247	$LiNi_{0.33}Mn_{0.33}Co_{0.33}O_2$	ICSD-171750
$LiNiO_2$	mp-25411	NiO_2	mp-25210
$LiCoO_2$	mp-22526	CuO	mp-704645
CeO_2	mp-20194	RuO_2	mp-825
HfO_2	mp-352	MgO	mp-1265
ZnO	mp-2133	SnO_2	mp-856
Fe_2O_3	mp-19770	Y_2O_3	mp-2652
$ZnAl_2O_4$	mp-2908	ZrO_2	mp-2858
Al_2O_3	mp-1143	TiO_2	mp-2657
SiO_2	mp-6930	Li_2O	mp-1960
Li_3BO_3	mp-27275	$LiTaO_3$	mp-3666
Li_2ZrO_3	mp-4156	Li_2WO_4	mp-18933
Li_2MoO_4	mp-1202737	$Li_3B_{11}O_{18}$	mp-1020014
$LiB(C_2O_4)_2$	mp-556165	Li_2TiO_3	mp-2931
$Li_{0.5}La_{0.5}TiO_3$	Ref.[21]	Li_2SiO_3	mp-5012
Li_3NbO_4	mp-31488	Li_3VO_4	mp-19219
$LiNbO_3$	mp-3731	$Li_4Ti_5O_{12}$	Ref.[22]
$LiAlO_2$	mp-3427	Li_2PO_2N	mp-1020019
$LiNO_3$	mp-8180	Li_4SiO_4	mp-559848
AlF_3	mp-468	MgF_2	mp-1249
ZrF_4	mp-1194272	FeF_3	mp-22398
LaF_3	mp-8354	BaF_2	mp-1029
ZnF_2	mp-1873	NiF_2	mp-559798
YF_3	mp-2416	PrF_3	mp-2651
SmF_3	mp-7384	CaF_2	mp-2741

CeF_4	mp-542001	LiF	mp-1138
LiCl	mp-22905	LiBr	mp-23259
LiI	mp-22899	$Li_{1.4}Al_{0.4}Ti_{1.6}(PO_4)_3$	Ref.[23]
$Li_{3.5}Si_{0.5}P_{0.5}O_4$	Ref.[24]	$AlPO_4$	mp-5331
$Mn_3(PO_4)_2$	mp-558631	$Co_3(PO_4)_2$	mp-19264
$Ni_3(PO_4)_2$	mp-22697	$LaPO_4$	mp-3962
YPO_4	mp-5132	$TiPO_4$	mp-5839
Li_3PO_4	mp-13725	LiH_2PO_4	mp-24610
Li_3BS_3	mp-5614	ZnS	mp-10695
CdS	mp-672	Sc_2S_3	mp-401
La_2S_3	mp-7475	SiS_2	mp-1602
TiS_2	mp-2156	ZrS_2	mp-1186
HfS_2	mp-985829	Li_2Se	mp-2286
TiN	mp-492	AlN	mp-661
Li_2CO_3	mp-3054		

Table S1: Sources of compounds considered in this work.

2 Interfacial stability

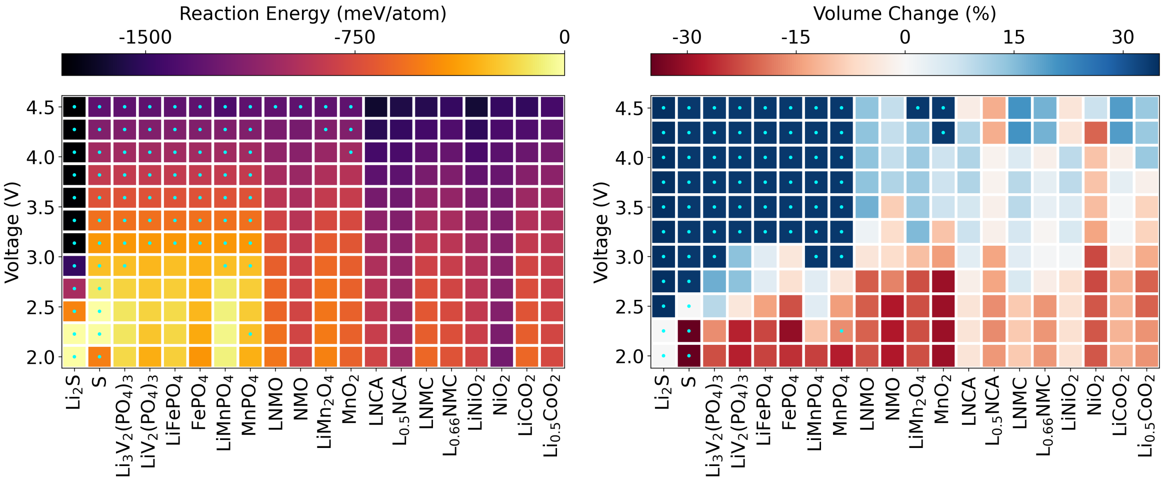

Figure S3: Electrochemical reaction energy (left) and corresponding volume change (right) associated with reactions at various cathode/Li_3PS_4 interfaces calculated using DFT with GGA density functional and GGA+U approach. The dot represents the decomposition of the material at the interface, indicating the material decomposes with a more negative reaction energy than that of the interfacial reaction.

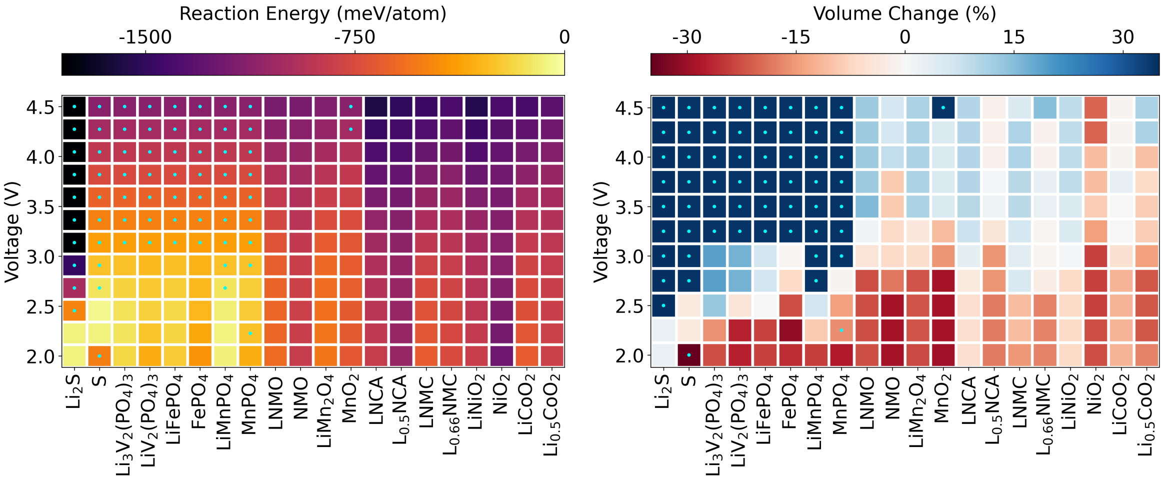

Figure S4: Electrochemical reaction energy (left) and corresponding volume change (right) associated with reactions at various cathode/$Li_7P_3S_{11}$ interfaces calculated using DFT with GGA density functional and GGA+U approach. The dot represents the decomposition of the material at the interface, indicating the material decomposes with a more negative reaction energy than that of the interfacial reaction.

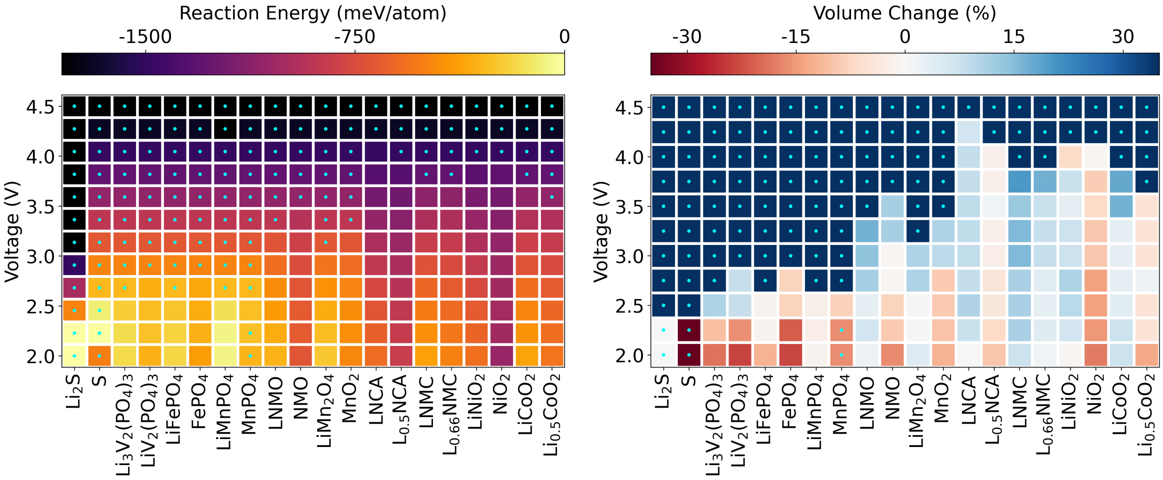

Figure S5: Electrochemical reaction energy (left) and corresponding volume change (right) associated with reactions at various cathode/Li_4GeS_4 interfaces calculated using DFT with GGA density functional and GGA+U approach. The dot represents the decomposition of the material at the interface, indicating the material decomposes with a more negative reaction energy than that of the interfacial reaction.

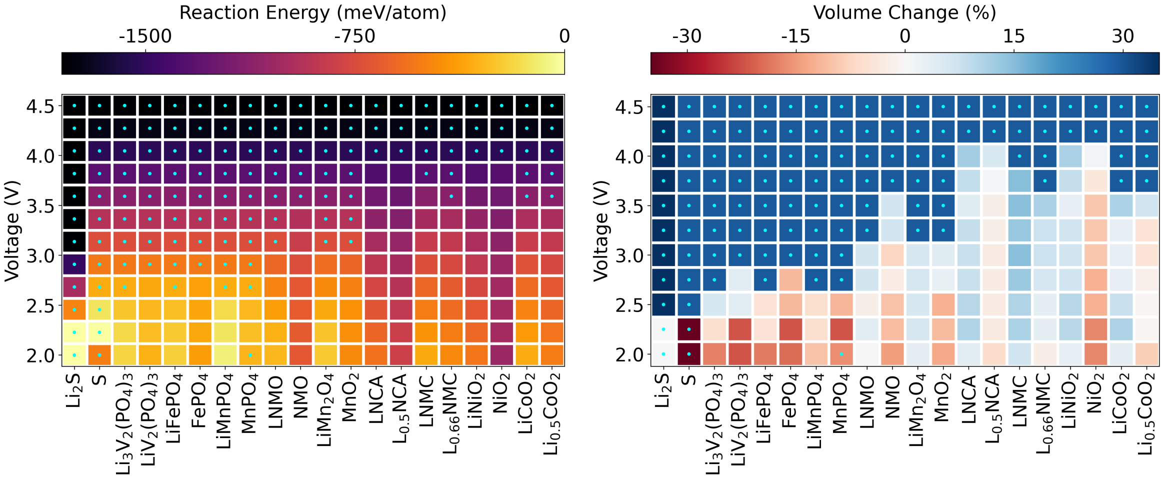

Figure S6: Electrochemical reaction energy (left) and corresponding volume change (right) associated with reactions at various cathode/Li_4SnS_4 interfaces calculated using DFT with GGA density functional and GGA+U approach. The dot represents the decomposition of the material at the interface, indicating the material decomposes with a more negative reaction energy than that of the interfacial reaction.

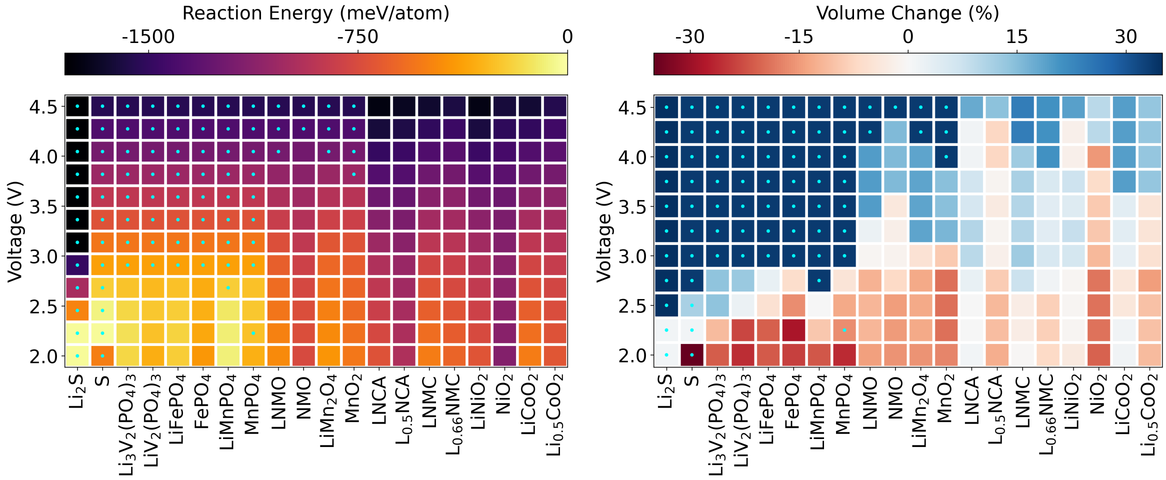

Figure S7: Electrochemical reaction energy (left) and corresponding volume change (right) associated with reactions at various cathode/$Li_{10}SnP_2S_{12}$ interfaces calculated using DFT with GGA density functional and GGA+U approach. The dot represents the decomposition of the material at the interface, indicating the material decomposes with a more negative reaction energy than that of the interfacial reaction.

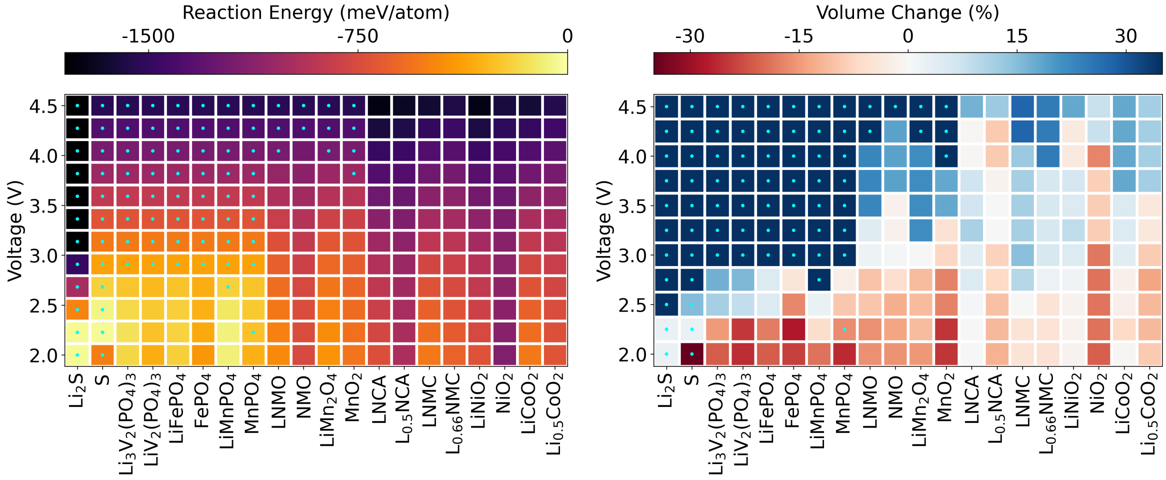

Figure S8: Electrochemical reaction energy (left) and corresponding volume change (right) associated with reactions at various cathode/$Li_{10}GeP_2S_{12}$ interfaces calculated using DFT with GGA density functional and GGA+U approach. The dot represents the decomposition of the material at the interface, indicating the material decomposes with a more negative reaction energy than that of the interfacial reaction.

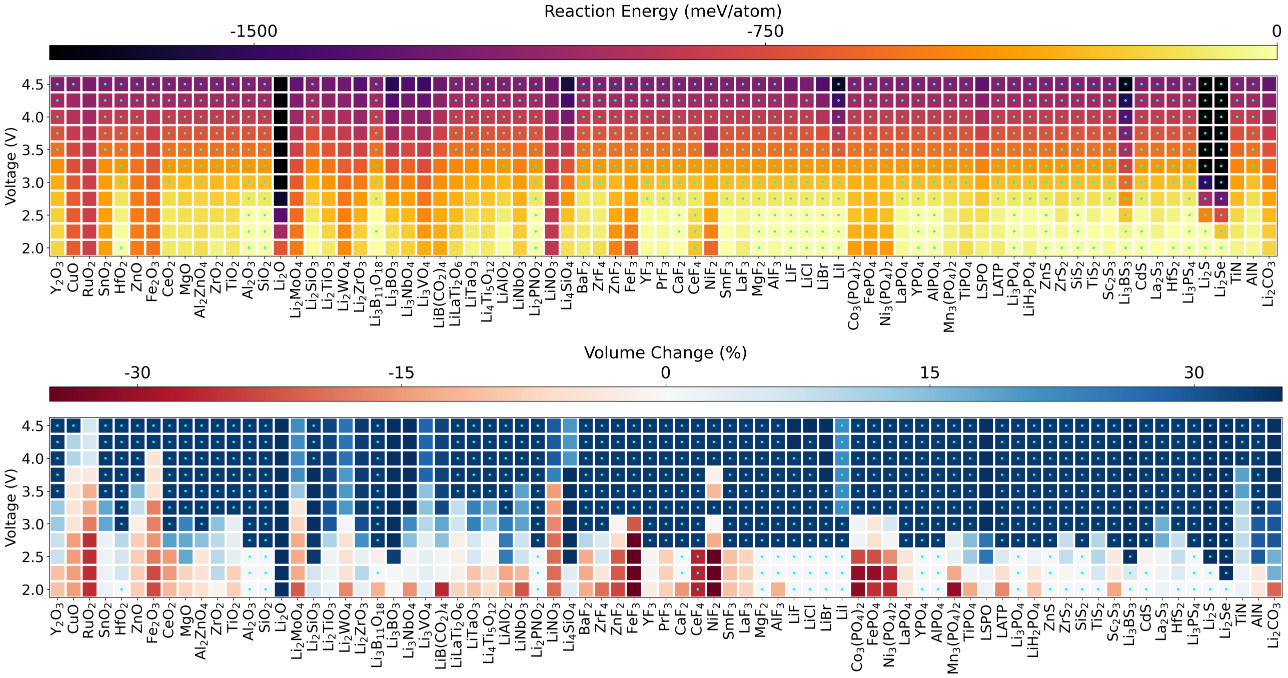

Figure S9: Electrochemical reaction energy (top) and corresponding volume change (bottom) associated with reactions at various Li_3PS_4/interlayer and Li_3PS_4/coating interfaces calculated using DFT with GGA density functional and GGA+U approach. The dot represents the decomposition of the material at the interface, indicating the material decomposes with a more negative reaction energy than that of the interfacial reaction.

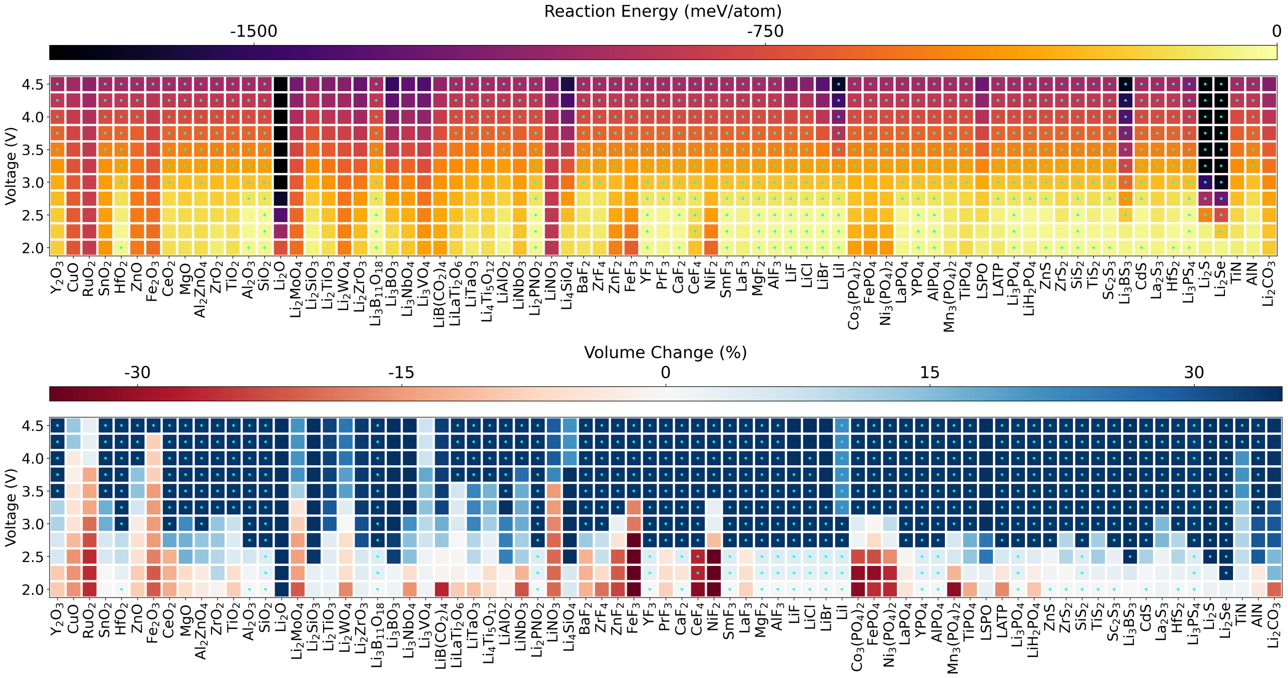

Figure S10: Electrochemical reaction energy (top) and corresponding volume change (bottom) associated with reactions at various $Li_7P_3S_{11}$/interlayer and $Li_7P_3S_{11}$/coating interfaces calculated using DFT with GGA density functional and GGA+U approach. The dot represents the decomposition of the material at the interface, indicating the material decomposes with a more negative reaction energy than that of the interfacial reaction.

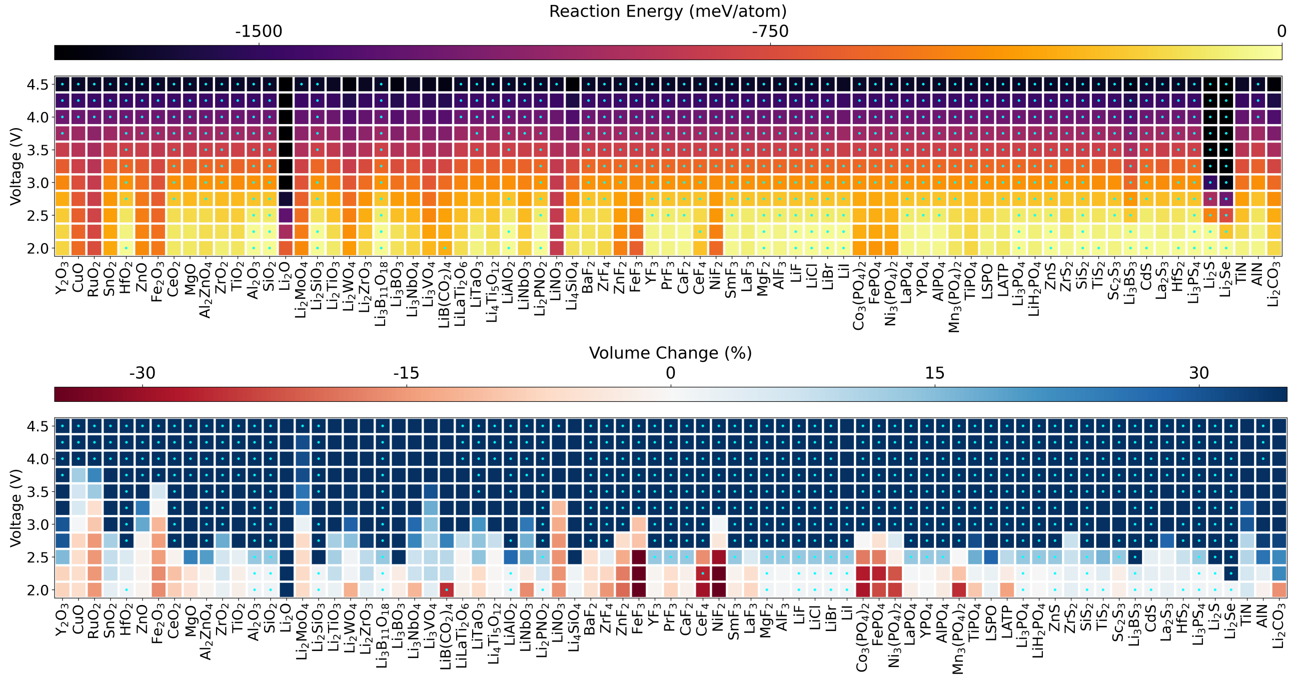

Figure S11: Electrochemical reaction energy (top) and corresponding volume change (bottom) associated with reactions at various Li_6PS_5Cl/interlayer and Li_6PS_5Cl/coating interfaces calculated using DFT with GGA density functional and GGA+U approach. The dot represents the decomposition of the material at the interface, indicating the material decomposes with a more negative reaction energy than that of the interfacial reaction.

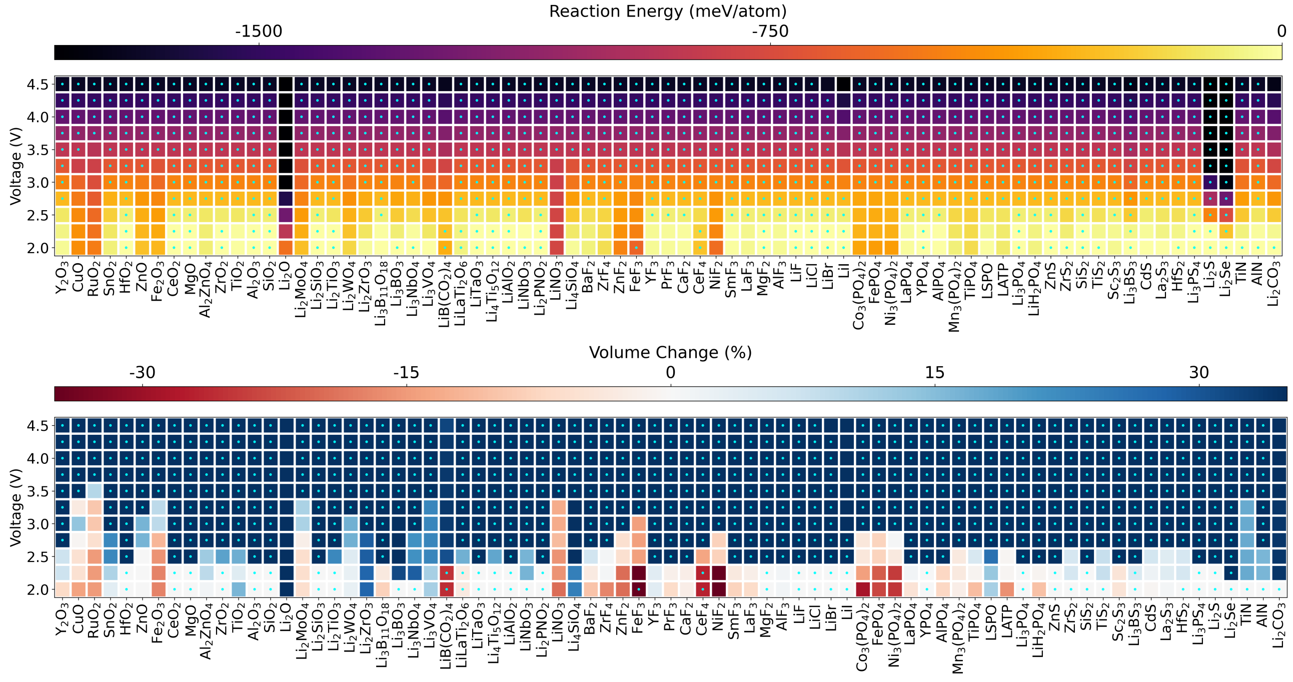

Figure S12: Electrochemical reaction energy (top) and corresponding volume change (bottom) associated with reactions at various Li_4GeS_4/interlayer and Li_4GeS_4/coating interfaces calculated using DFT with GGA density functional and GGA+U approach. The dot represents the decomposition of the material at the interface, indicating the material decomposes with a more negative reaction energy than that of the interfacial reaction.

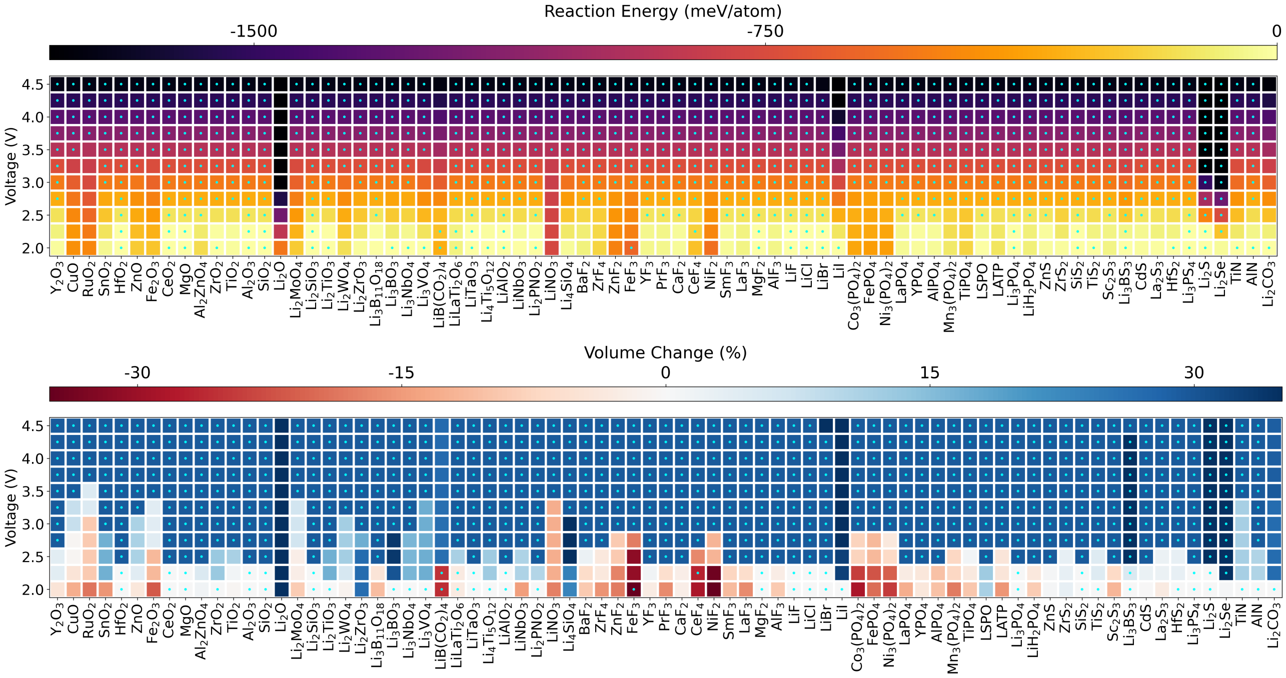

Figure S13: Electrochemical reaction energy (top) and corresponding volume change (bottom) associated with reactions at various Li_4SnS_4/interlayer and Li_4SnS_4/coating interfaces calculated using DFT with GGA density functional and GGA+U approach. The dot represents the decomposition of the material at the interface, indicating the material decomposes with a more negative reaction energy than that of the interfacial reaction.

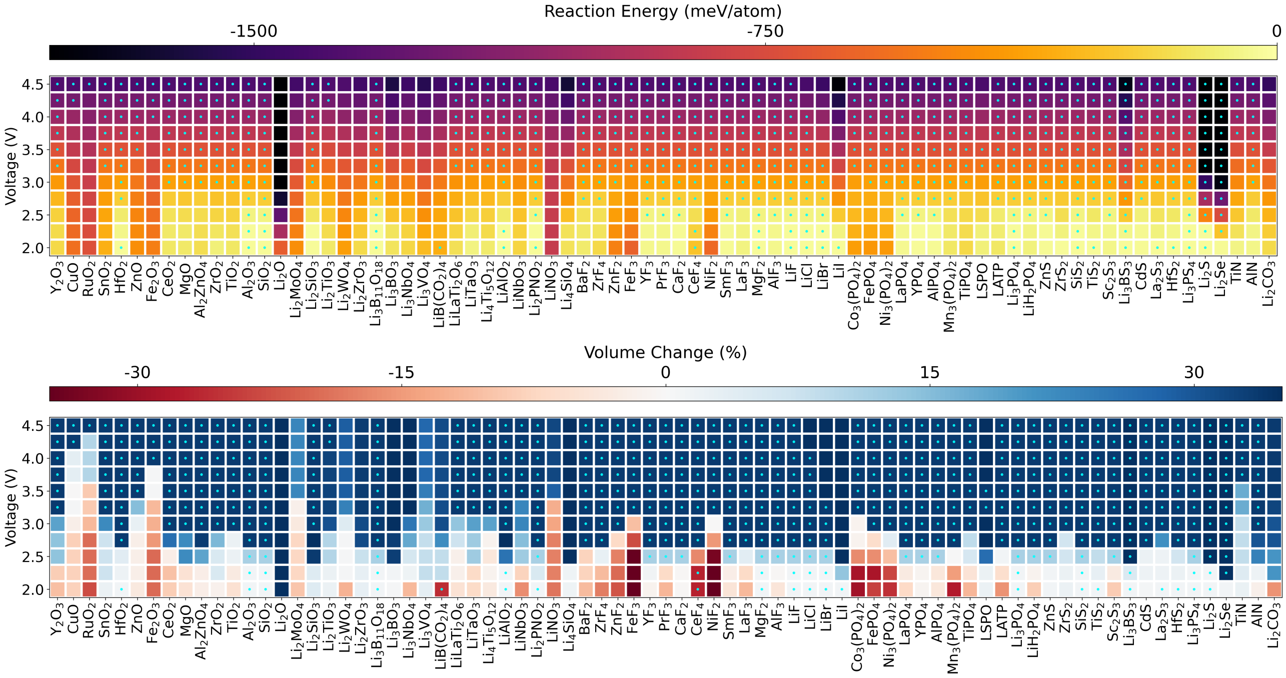

Figure S14: Electrochemical reaction energy (top) and corresponding volume change (bottom) associated with reactions at various $Li_{10}SnP_2S_{12}$/interlayer and $Li_{10}SnP_2S_{12}$/coating interfaces calculated using DFT with GGA density functional and GGA+U approach. The dot represents the decomposition of the material at the interface, indicating the material decomposes with a more negative reaction energy than that of the interfacial reaction.

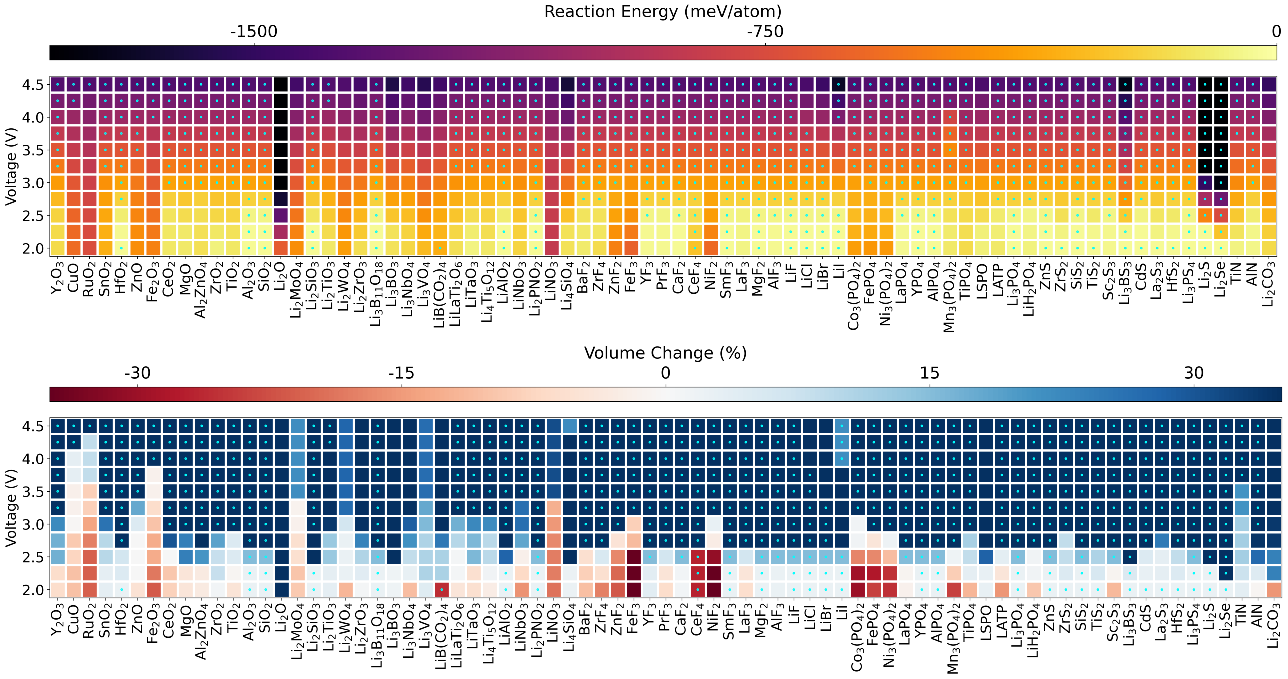

Figure S15: Electrochemical reaction energy (top) and corresponding volume change (bottom) associated with reactions at various $Li_{10}GeP_2S_{12}$/interlayer and $Li_{10}GeP_2S_{12}$/coating interfaces calculated using DFT with GGA density functional and GGA+U approach. The dot represents the decomposition of the material at the interface, indicating the material decomposes with a more negative reaction energy than that of the interfacial reaction.

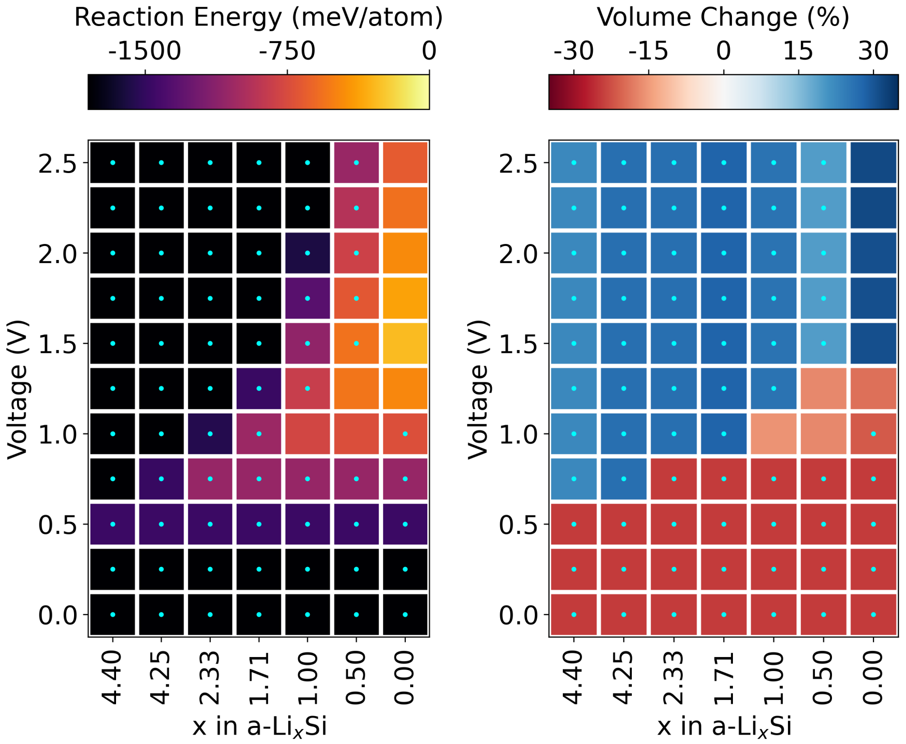

Figure S16: Electrochemical reaction energy (left) and corresponding volume change (right) associated with reactions at various a-Li_xSi/Li_3PS_4 interfaces calculated using DFT with GGA density functional and GGA+U approach. The dot represents the decomposition of the material at the interface, indicating the material decomposes with a more negative reaction energy than that of the interfacial reaction.

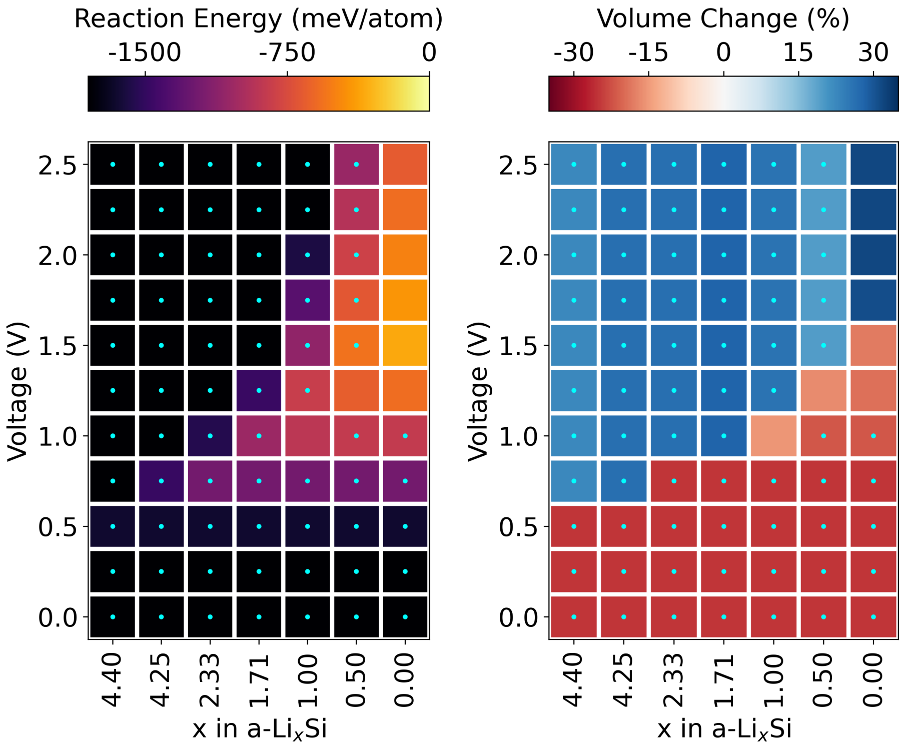

Figure S17: Electrochemical reaction energy (left) and corresponding volume change (right) associated with reactions at various a-Li_xSi/Li_7PS_{11} interfaces calculated using DFT with GGA density functional and GGA+U approach. The dot represents the decomposition of the material at the interface, indicating the material decomposes with a more negative reaction energy than that of the interfacial reaction.

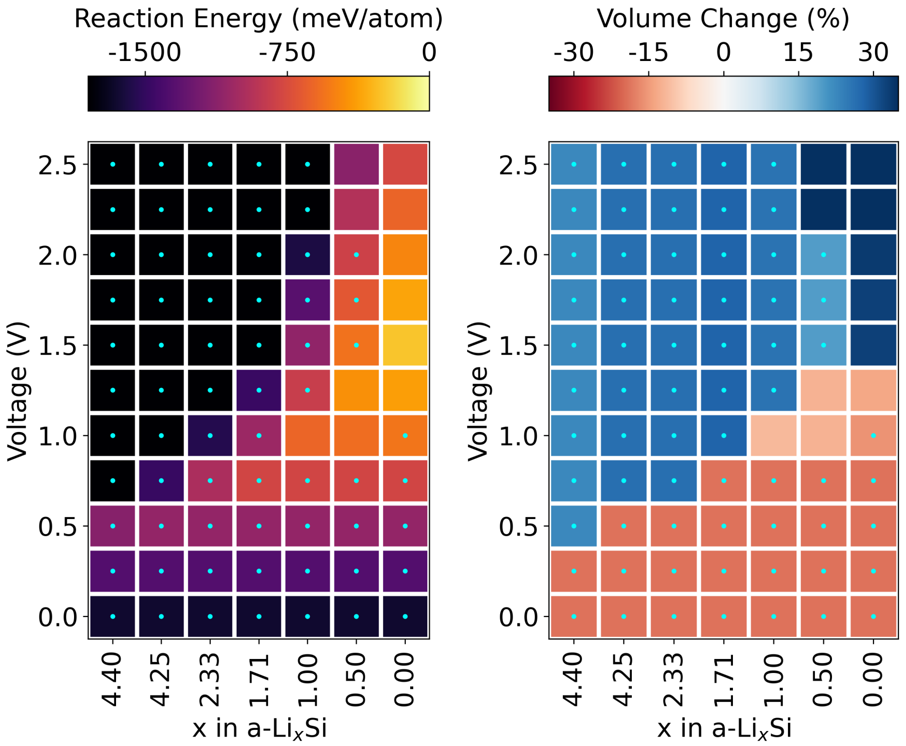

Figure S18: Electrochemical reaction energy (left) and corresponding volume change (right) associated with reactions at various a-Li_xSi/Li_6PS_5Cl interfaces calculated using DFT with GGA density functional and GGA+U approach. The dot represents the decomposition of the material at the interface, indicating the material decomposes with a more negative reaction energy than that of the interfacial reaction.

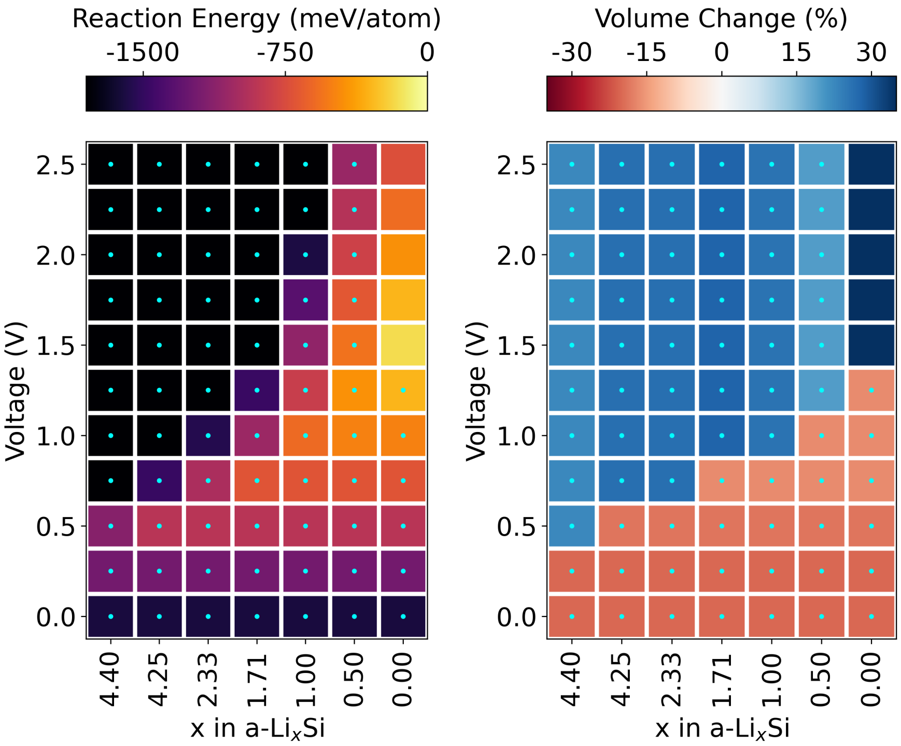

Figure S19: Electrochemical reaction energy (left) and corresponding volume change (right) associated with reactions at various a-Li_xSi/Li_4GeS_4 interfaces calculated using DFT with GGA density functional and GGA+U approach. The dot represents the decomposition of the material at the interface, indicating the material decomposes with a more negative reaction energy than that of the interfacial reaction.

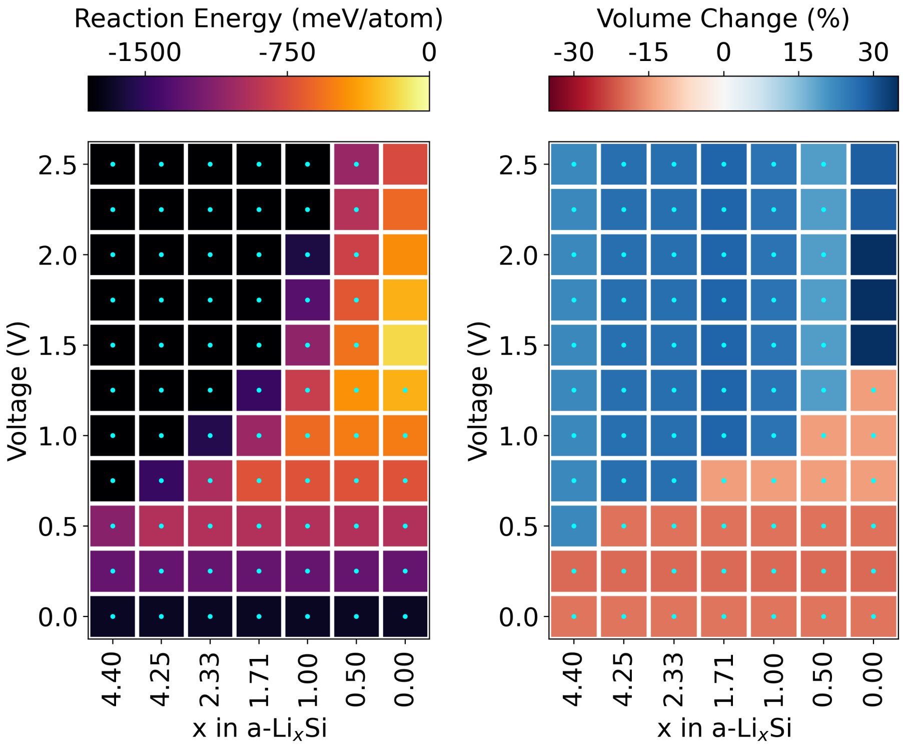

Figure S20: Electrochemical reaction energy (left) and corresponding volume change (right) associated with reactions at various a-Li_xSi/Li_4SnS_4 interfaces calculated using DFT with GGA density functional and GGA+U approach. The dot represents the decomposition of the material at the interface, indicating the material decomposes with a more negative reaction energy than that of the interfacial reaction.

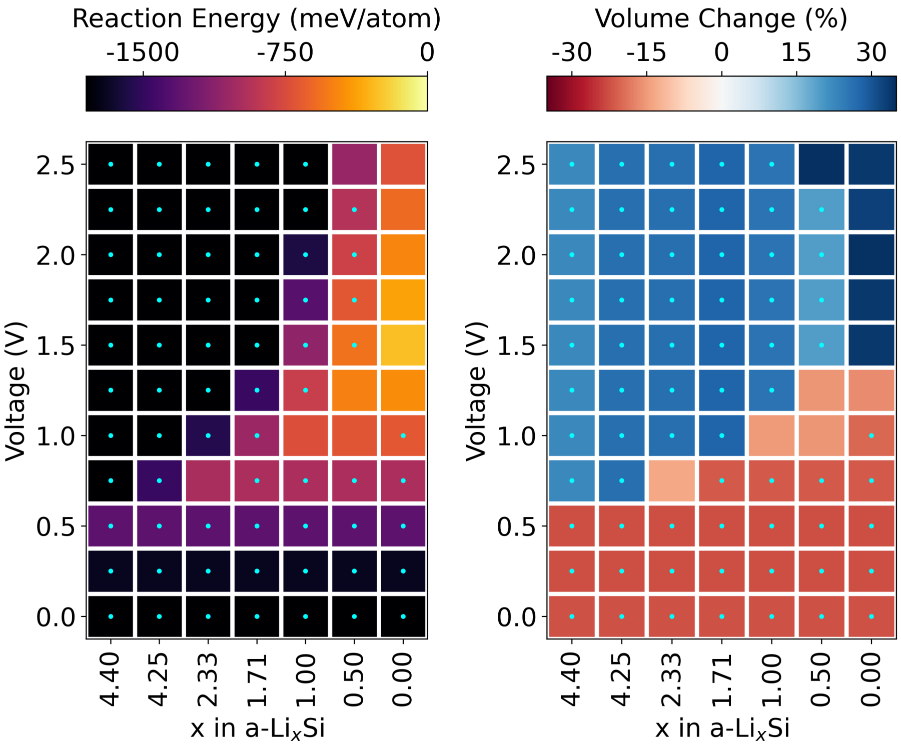

Figure S21: Electrochemical reaction energy (left) and corresponding volume change (right) associated with reactions at various a-Li_xSi/$Li_{10}SnP_2S_{12}$ interfaces calculated using DFT with GGA density functional and GGA+U approach. The dot represents the decomposition of the material at the interface, indicating the material decomposes with a more negative reaction energy than that of the interfacial reaction.

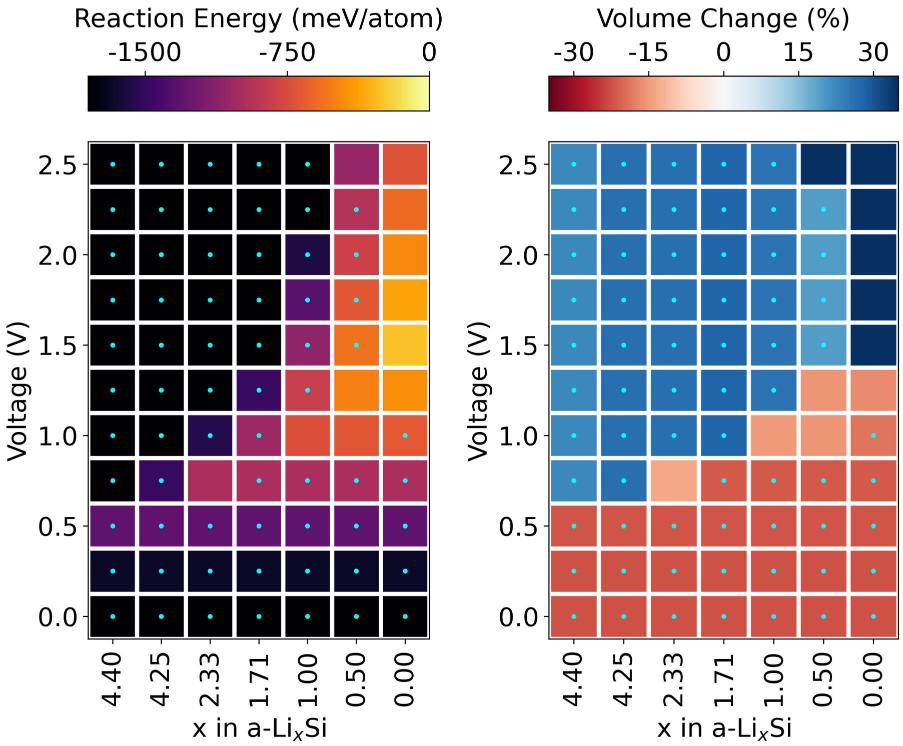

Figure S22: Electrochemical reaction energy (left) and corresponding volume change (right) associated with reactions at various a-Li_xSi/$Li_{10}GeP_2S_{12}$ interfaces calculated using DFT with GGA density functional and GGA+U approach. The dot represents the decomposition of the material at the interface, indicating the material decomposes with a more negative reaction energy than that of the interfacial reaction.

3 Kinetics

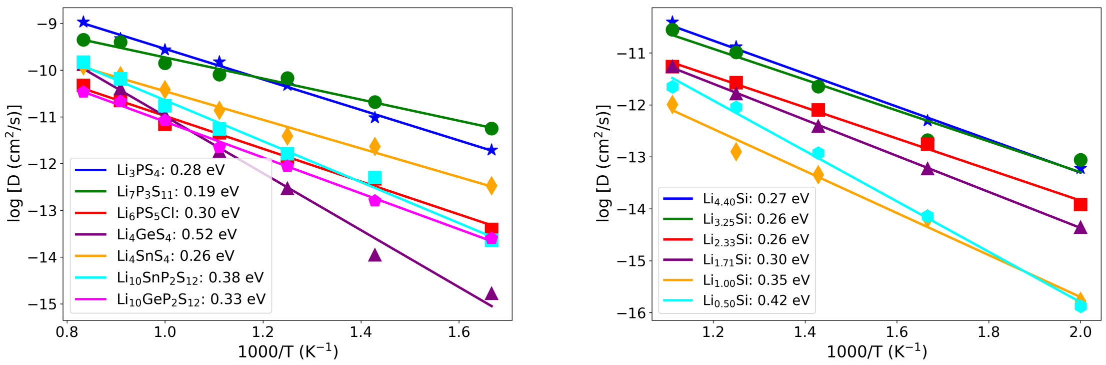

Figure S23: Arrhenius plots for SE and a-Li_xSi bulk. The bulk activation energy E_a is shown.

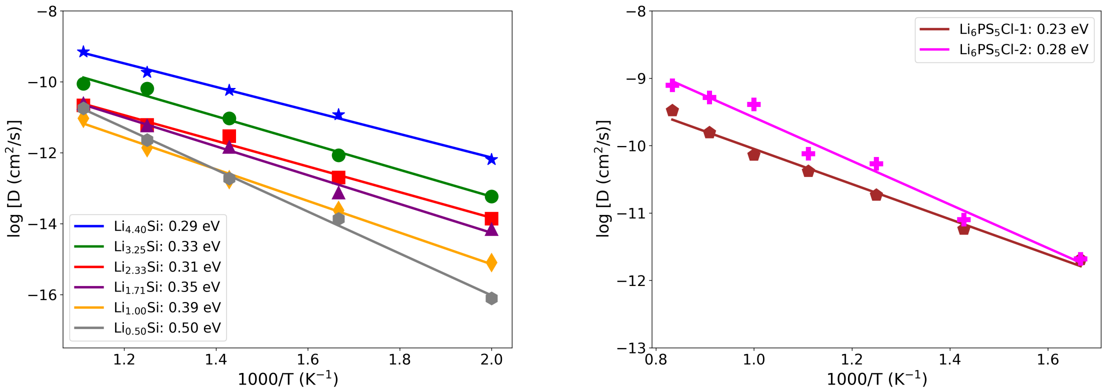

Figure S24: Arrhenius plots for a-Li_xSi and Li_6PS_5Cl slab. The surface activation energy E_a is shown.

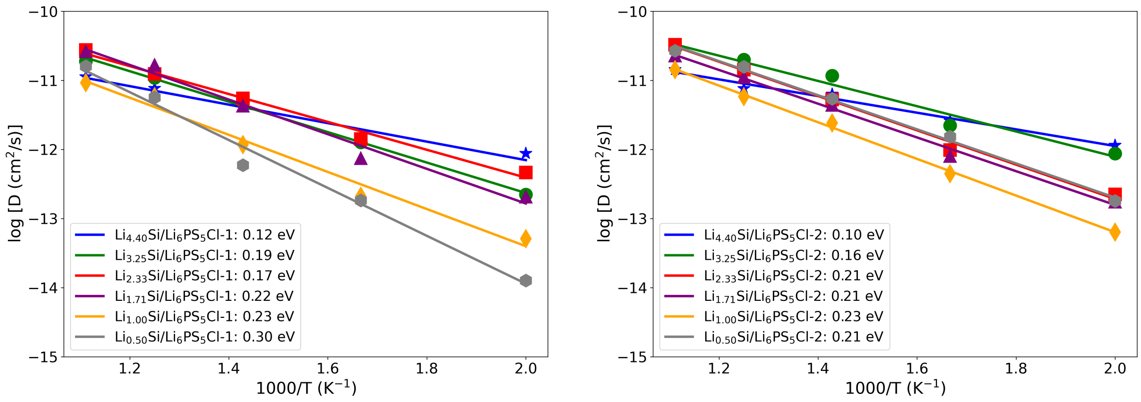

Figure S25: Arrhenius plots for a-Li_xSi/Li_6PS_5Cl interface. The interface activation energy E_a is shown.

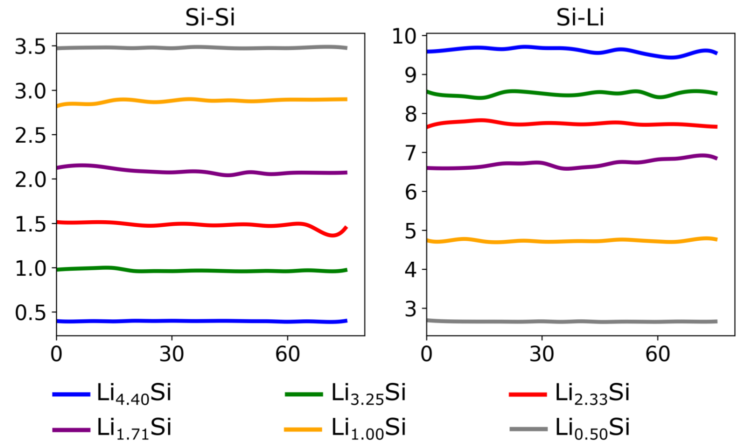

Figure S26: Evolution of the average coordination number of a-Li_xSi bulk at 298 K. Averaging is carried out over elements as well as over time with a time step of 5 ps.

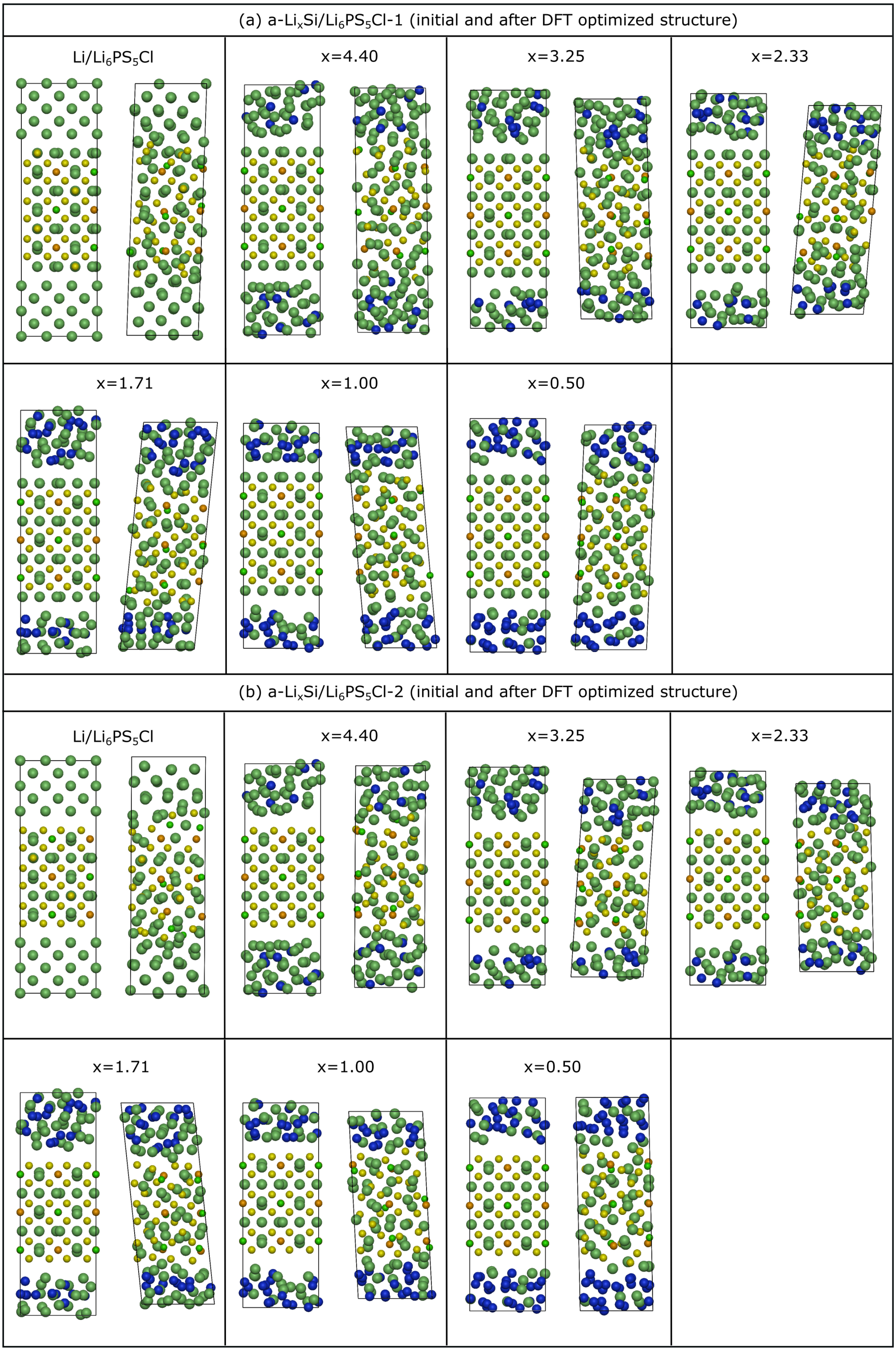

Figure S27: Atomic structure of initial and after DFT optimized a-Li_xSi/Li_6PS_5Cl-1 and a-Li_xSi/Li_6PS_5Cl-2 interfaces.

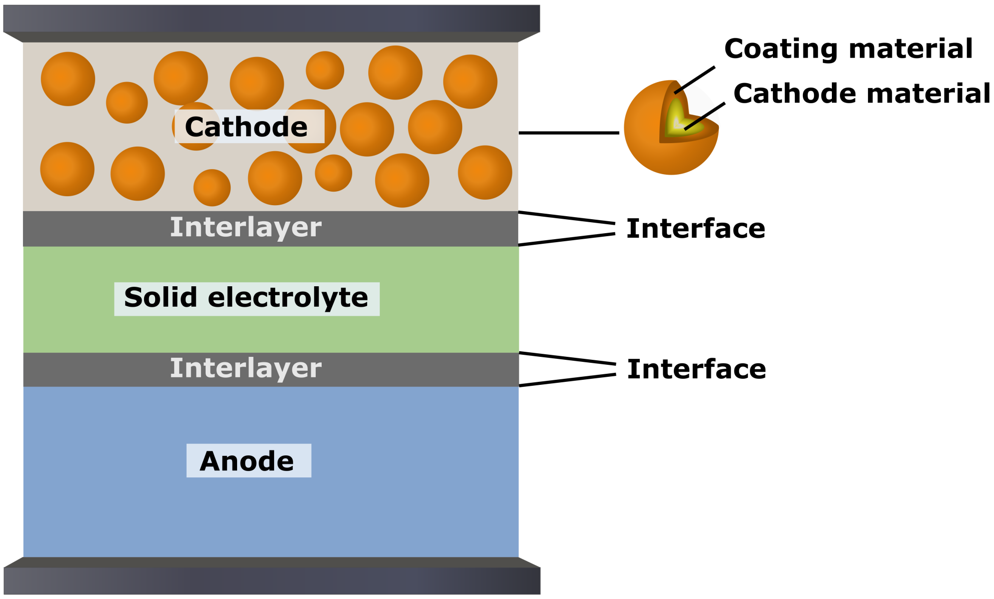

Figure S27: Schematic illustration of interfaces in ASSBs. The interlayer at the cathode side, often formed through reaction between cathode and solid electrolytes, is often denoted as "cathode electrolyte interphase". The interlayer between the anode and the solid electrolyte is often denoted as "solid electrolyte interlayer".

**Reactants**	**Products**
$Li_3V_2(PO_4)_3$, Li_3PS_4	P_4S_7, $Li_4P_2O_7$, P_4S_3, VS_2
$LiV_2(PO_4)_3$, Li_3PS_4	VS_2, $LiPO_3$
$LiFePO_4$, Li_3PS_4	$Li_4P_2O_7$, FePS, FeS_2
$FePO_4$, Li_3PS_4	FePS, $Li_4P_2O_7$, $LiPO_3$, FeS_2
$LiMnPO_4$, Li_3PS_4	$MnPS_3$, Li_3PO_4, MnS_2
$MnPO_4$, Li_3PS_4	$Mn_2P_2O_7$, $LiPO_3$, MnS_2
$LiNi_{0.5}Mn_{1.5}O_4$, Li_3PS_4	Li_3PO_4, Ni_3S_4, MnO, MnS_2, Li_2S
$Ni_{0.5}Mn_{1.5}O_4$, Li_3PS_4	$Li_2Mn(SO_4)_2$, $Mn_3(PO_4)_2$, Ni_3S_4, MnO, Li_2SO_4
$LiMn_2O_4$, Li_3PS_4	MnS, Li_3PO_4, MnS_2, Li_2S
MnO_2, Li_3PS_4	Li_3PO_4, MnS_2
$LiNi_{0.8}Co_{0.15}Al_{0.05}O_2$, Li_3PS_4	Ni_3S_2, Li_3PO_4, $LiAlO_2$, Co_2NiS_4, Li_2SO_4, Li_2S
$Li_{0.5}Ni_{0.8}Co_{0.15}Al_{0.05}O_2$, Li_3PS_4	Ni_3S_2, Li_3PO_4, Co_9S_8, Co_2NiS_4, Li_2SO_4, Al_2O_3
$LiNi_{0.33}Mn_{0.33}Co_{0.33}O_2$, Li_3PS_4	Ni_3S_2, Co_2NiS_4, Li_3PO_4, Ni_3S_4, MnS_2, Li_2S
$Li_{0.67}Ni_{0.33}Mn_{0.33}Co_{0.33}O_2$, Li_3PS_4	Co_2NiS_4, MnO, Li_3PO_4, Ni_3S_4, Li_2S, Li_2SO_4
$LiNiO_2$, Li_3PS_4	Ni_3S_4, Li_2SO_4, Li_3PO_4, Li_2S
NiO_2, Li_3PS_4	$Ni_3(PO_4)_2$, Ni_3S_2, Li_2SO_4, $NiSO_4$
$LiCoO_2$, Li_3PS_4	Li_3PO_4, Li_2SO_4, Co_3S_4, Li_2S
$Li_{0.5}CoO_2$, Li_3PS_4	Co_9S_8, Li_3PO_4, Li_2SO_4, Li_2S
Li_2S, $Li_7P_3S_{11}$	Li_3PS_4
$Li_3V_2(PO_4)_3$, $Li_7P_3S_{11}$	P_4S_7, VS_2, P_4S_3, $Li_4P_2O_7$
$LiV_2(PO_4)_3$, $Li_7P_3S_{11}$	P_4S_9, VS_2, V_2PS_{10}, $LiPO_3$
$LiFePO_4$, $Li_7P_3S_{11}$	$Li_4P_2O_7$, FePS, $LiPO_3$, FeS_2
$Li_7P_3S_{11}$, $FePO_4$	P_4S_7, FePS, $LiPO_3$, FeS_2
$LiMnPO_4$, $Li_7P_3S_{11}$	P_4S_9, $MnPS_3$, Li_3PO_4, MnS_2
$MnPO_4$, $Li_7P_3S_{11}$	$Mn_2P_2O_7$, $Mn(PO_3)_2$, $LiPO_3$, MnS_2
$LiNi_{0.5}Mn_{1.5}O_4$, $Li_7P_3S_{11}$	Ni_3S_2, Li_3PO_4, Ni_3S_4, MnS_2, Li_2S
$Ni_{0.5}Mn_{1.5}O_4$, $Li_7P_3S_{11}$	$Li_2Mn(SO_4)_2$, $Mn_3(PO_4)_2$, MnO, Li_2SO_4, Ni_3S_4
$LiMn_2O_4$, $Li_7P_3S_{11}$	MnS, Li_3PO_4, MnS_2, Li_2S
MnO_2, $Li_7P_3S_{11}$	$LiMnPO_4$, Li_3PO_4, MnS_2, Li_2SO_4
$LiNi_{0.8}Co_{0.15}Al_{0.05}O_2$, $Li_7P_3S_{11}$	Li_3PO_4, $LiAlO_2$, Ni_3S_4, Co_2NiS_4, Li_2SO_4, Li_2S
$Li_{0.5}Ni_{0.8}Co_{0.15}Al_{0.05}O_2$, $Li_7P_3S_{11}$	Ni_3S_2, Li_3PO_4, Ni_3S_4, Co_2NiS_4, Li_2SO_4, Al_2O_3
$LiNi_{0.33}Mn_{0.33}Co_{0.33}O_2$, $Li_7P_3S_{11}$	Ni_3S_2, Co_2NiS_4, Li_3PO_4, Ni_3S_4, MnS_2, Li_2S
$Li_{0.67}Ni_{0.33}Mn_{0.33}Co_{0.33}O_2$, $Li_7P_3S_{11}$	Co_2NiS_4, Li_3PO_4, Ni_3S_4, MnS_2, Li_2S, Li_2SO_4

$LiNiO_2$, $Li_7P_3S_{11}$	Ni_3S_4, Li_2SO_4, Li_3PO_4, Li_2S
NiO_2, $Li_7P_3S_{11}$	$NiSO_4$, Ni_3S_2, $Ni_3(PO_4)_2$, Li_2SO_4
$LiCoO_2$, $Li_7P_3S_{11}$	Li_3PO_4, CoS_2, Co_3S_4, Li_2S
$Li_{0.5}CoO_2$, $Li_7P_3S_{11}$	Ni_3S_2, Li_3PO_4, Co_2NiS_4, Li_2SO_4, Co_3S_4
$Li_3V_2(PO_4)_3$, Li_6PS_5Cl	P_4S_7, VS_2, P_4S_3, LiCl, Li_3PO_4
$LiV_2(PO_4)_3$, Li_6PS_5Cl	V_2PS_{10}, P_4S_9, VS_2, $Li_4P_2O_7$, LiCl
$LiFePO_4$, Li_6PS_5Cl	FePS, Li_3PO_4, $Li_4P_2O_7$, FeS_2, LiCl
$FePO_4$, Li_6PS_5Cl	FePS, $LiPO_3$, $Li_4P_2O_7$, FeS_2, LiCl
$LiMnPO_4$, Li_6PS_5Cl	$MnPS_3$, MnP_4, Li_3PO_4, MnS_2, LiCl
$MnPO_4$, Li_6PS_5Cl	$Li_2MnP_2O_7$, $Li_4P_2O_7$, Mn_2PClO_4, $LiPO_3$, MnS_2
$LiNi_{0.5}Mn_{1.5}O_4$, Li_6PS_5Cl	LiCl, Li_2S, MnO, Ni_3S_4, MnS_2, Li_3PO_4
$Ni_{0.5}Mn_{1.5}O_4$, Li_6PS_5Cl	Ni_3S_2, Li_2SO_4, $Li_2Mn(SO_4)_2$, MnO, Mn_2PClO_4
$LiMn_2O_4$, Li_6PS_5Cl	MnO, LiCl, Li_2S, MnS_2, Li_3PO_4
MnO_2, Li_6PS_5Cl	Li_2SO_4, Mn_2PClO_4, MnO, MnS_2
$LiNi_{0.8}Co_{0.15}Al_{0.05}O_2$, Li_6PS_5Cl	Ni_3S_2, Li_2SO_4, Li_5AlO_4, Co_9S_8, Li_3PO_4, Li_2S, LiCl
$Li_{0.5}Ni_{0.8}Co_{0.15}Al_{0.05}O_2$, Li_6PS_5Cl	Ni_3S_2, Li_2SO_4, Co_9S_8, $LiAlO_2$, Li_3PO_4, LiCl, Ni
$LiNi_{0.33}Mn_{0.33}Co_{0.33}O_2$, Li_6PS_5Cl	Li_2SO_4, Ni_3S_2, MnO, Co_2NiS_4, Li_2S, LiCl, Li_3PO_4
$Li_{0.67}Ni_{0.33}Mn_{0.33}Co_{0.33}O_2$, Li_6PS_5Cl	Li_2SO_4, Ni_3S_2, Co_9S_8, $LiMnO_2$, Li_2S, LiCl, Li_3PO_4
$LiNiO_2$, Li_6PS_5Cl	Li_2S, Li_3PO_4, Ni_3S_2, LiCl, Li_2SO_4
NiO_2, Li_6PS_5Cl	Ni_3S_2, $Ni_3(PO_4)_2$, NiO, $NiCl_2$, Li_2SO_4
$LiCoO_2$, Li_6PS_5Cl	Li_2S, Li_3PO_4, Co_9S_8, LiCl, Li_2SO_4
$Li_{0.5}CoO_2$, Li_6PS_5Cl	Li_3PO_4, Li_2SO_4, Co_9S_8, Li_2O, LiCl
$Li_3V_2(PO_4)_3$, Li_4GeS_4	V_4GeS_8, GeS_2, Li_3PO_4
$LiV_2(PO_4)_3$, Li_4GeS_4	GeS_2, VS_2, Li_3PO_4
$LiFePO_4$, Li_4GeS_4	FeS_2, Li_3PO_4, Ge
$FePO_4$, Li_4GeS_4	GeS, FeS_2, GeS_2, Li_3PO_4
$LiMnPO_4$, Li_4GeS_4	$Li_4MnGe_2S_7$, MnS, Li_3PO_4
$MnPO_4$, Li_4GeS_4	$LiMnPO_4$, GeS_2, MnS_2, Li_3PO_4
$LiNi_{0.5}Mn_{1.5}O_4$, Li_4GeS_4	Li_4GeO_4, $LiMnO_2$, Ni_3S_2, MnO, Li_2SO_4
$Ni_{0.5}Mn_{1.5}O_4$, Li_4GeS_4	$Li_2Mn(SO_4)_2$, Ni_3S_2, Mn_2GeO_4, Mn_3O_4, Li_2SO_4
$LiMn_2O_4$, Li_4GeS_4	$LiMnO_2$, Li_4GeO_4, MnO, Li_2SO_4
MnO_2, Li_4GeS_4	$Li_2Mn(SO_4)_2$, Mn_2GeO_4, MnO
$LiNi_{0.8}Co_{0.15}Al_{0.05}O_2$, Li_4GeS_4	Li_4GeO_4, Li_2O, Li_5AlO_4, Ni_3S_2, Li_2SO_4, Co_9S_8
$Li_{0.5}Ni_{0.8}Co_{0.15}Al_{0.05}O_2$, Li_4GeS_4	Li_3AlGeO_5, Li_2GeO_3, CoO, Li_2SO_4, Ni_2GeO_4, Ni

$LiNi_{0.33}Mn_{0.33}Co_{0.33}O_2$, Li_4GeS_4	$LiMnO_2$, Ni_3S_2, Li_4GeO_4, Co_9S_8, Li_2S, Li_2SO_4
$Li_{0.67}Ni_{0.33}Mn_{0.33}Co_{0.33}O_2$, Li_4GeS_4	$LiMnO_2$, Ni_3S_2, Li_4GeO_4, Co_9S_8, Li_2SO_4, Ni
$LiNiO_2$, Li_4GeS_4	Ni_3S_2, Li_2SO_4, Li_2O, Li_4GeO_4
NiO_2, Li_4GeS_4	Ni_2GeO_4, Li_2SO_4, NiO, $NiSO_4$
$LiCoO_2$, Li_4GeS_4	Li_4GeO_4, Li_2S, Co_9S_8, Li_2SO_4
$Li_{0.5}CoO_2$, Li_4GeS_4	Li_4GeO_4, Co_9S_8, Li_2O, Li_2SO_4
$Li_3V_2(PO_4)_3$, Li_4SnS_4	SnS, SnS_2, VS_2, Li_3PO_4
$LiV_2(PO_4)_3$, Li_4SnS_4	SnS_2, VS_2, Li_3PO_4
$LiFePO_4$, Li_4SnS_4	SnS, Li_3PO_4, FeS_2, FeS
$FePO_4$, Li_4SnS_4	SnS, Li_3PO_4, FeS_2, SnS_2
$LiMnPO_4$, Li_4SnS_4	Li_2MnSnS_4, Li_3PO_4
$MnPO_4$, Li_4SnS_4	$LiMnPO_4$, SnS_2, MnS_2, Li_3PO_4
$LiNi_{0.5}Mn_{1.5}O_4$, Li_4SnS_4	$LiMnO_2$, Li_2SnO_3, MnO, Ni_3S_2, Li_2SO_4
$Ni_{0.5}Mn_{1.5}O_4$, Li_4SnS_4	$MnSnO_3$, $Li_2Mn(SO_4)_2$, Ni_3S_2, Mn_3O_4, Li_2SO_4
$LiMn_2O_4$, Li_4SnS_4	$LiMnO_2$, MnO, Li_2SnO_3, Li_2SO_4
MnO_2, Li_4SnS_4	$Li_2Mn(SO_4)_2$, $MnSnO_3$, MnO
$LiNi_{0.8}Co_{0.15}Al_{0.05}O_2$, Li_4SnS_4	Li_8SnO_6, Li_2SnO_3, Li_5AlO_4, Li_2SO_4, Ni, Co
$Li_{0.5}Ni_{0.8}Co_{0.15}Al_{0.05}O_2$, Li_4SnS_4	Li_2SnO_3, CoO, $LiAlO_2$, Li_2SO_4, SnO_2, Ni
$LiNi_{0.33}Mn_{0.33}Co_{0.33}O_2$, Li_4SnS_4	Co_9S_8, Ni_3S_2, $LiMnO_2$, Li_2SnO_3, Li_8SnO_6, Li_2SO_4
$Li_{0.67}Ni_{0.33}Mn_{0.33}Co_{0.33}O_2$, Li_4SnS_4	Co_9S_8, $LiMnO_2$, Li_2SnO_3, Li_8SnO_6, Li_2SO_4, Ni
$LiNiO_2$, Li_4SnS_4	Li_8SnO_6, Li_2SnO_3, Li_2SO_4, Ni
NiO_2, Li_4SnS_4	Li_2SO_4, NiO, $NiSO_4$, SnO_2
$LiCoO_2$, Li_4SnS_4	Li_8SnO_6, Co_9S_8, Li_2SnO_3, Li_2SO_4
$Li_{0.5}CoO_2$, Li_4SnS_4	Co_9S_8, Li_2SnO_3, Li_2SO_4, Co
$Li_3V_2(PO_4)_3$, $Li_{10}SnP_2S_{12}$	P_4S_7, $SnPS_3$, $Li_4P_2O_7$, VS_2, Li_3PO_4
$LiV_2(PO_4)_3$, $Li_{10}SnP_2S_{12}$	VS_4, $SnPS_3$, $Li_4P_2O_7$, $LiPO_3$, VS_2
$LiFePO_4$, $Li_{10}SnP_2S_{12}$	$SnPS_3$, FePS, Li_3PO_4, FeS_2
$FePO_4$, $Li_{10}SnP_2S_{12}$	$Li_4P_2O_7$, $LiPO_3$, SnS, FeS_2, FeP
$LiMnPO_4$, $Li_{10}SnP_2S_{12}$	$SnPS_3$, Li_3PO_4, MnP, $MnPS_3$, MnS_2
$MnPO_4$, $Li_{10}SnP_2S_{12}$	$Li_2MnP_2O_7$, $Mn_2P_2O_7$, SnS_2, $LiPO_3$, MnS_2
$LiNi_{0.5}Mn_{1.5}O_4$, $Li_{10}SnP_2S_{12}$	Li_2MnSnS_4, Ni_3S_4, Li_3PO_4, MnS_2, Li_2S, Li_2SO_4
$Ni_{0.5}Mn_{1.5}O_4$, $Li_{10}SnP_2S_{12}$	MnO, Ni_3S_4, $Mn_3(PO_4)_2$, $MnSnO_3$, Ni_3S_2, Li_2SO_4
$LiMn_2O_4$, $Li_{10}SnP_2S_{12}$	Li_3PO_4, Li_2MnSnS_4, MnS_2, Li_2S
MnO_2, $Li_{10}SnP_2S_{12}$	MnO, $MnSnO_3$, Li_3PO_4, MnS_2, Li_2SO_4

$LiNi_{0.8}Co_{0.15}Al_{0.05}O_2$, $Li_{10}SnP_2S_{12}$	Li_5AlO_4, Li_2S, Co_9S_8, Ni_3S_2, Li_2SnO_3, Li_3PO_4, Li_2SO_4
$Li_{0.5}Ni_{0.8}Co_{0.15}Al_{0.05}O_2$, $Li_{10}SnP_2S_{12}$	$LiAlO_2$, Co_9S_8, Ni_3S_2, Li_2SnO_3, Li_3PO_4, Li_2SO_4, Ni
$LiNi_{0.33}Mn_{0.33}Co_{0.33}O_2$, $Li_{10}SnP_2S_{12}$	Li_2SO_4, Ni_3S_4, Co_2NiS_4, MnO, Li_2SnO_3, Li_2S, Li_3PO_4
$Li_{0.67}Ni_{0.33}Mn_{0.33}Co_{0.33}O_2$, $Li_{10}SnP_2S_{12}$	Ni_3S_2, Li_2SO_4, $LiMnO_2$, Co_2NiS_4, Li_2SnO_3, Li_2S, Li_3PO_4
$LiNiO_2$, $Li_{10}SnP_2S_{12}$	Li_2S, Ni_3S_2, Li_2SnO_3, Li_3PO_4, Li_2SO_4
NiO_2, $Li_{10}SnP_2S_{12}$	$Ni_3(PO_4)_2$, Ni_3S_2, $LiNiPO_4$, SnO_2, Li_2SO_4
$LiCoO_2$, $Li_{10}SnP_2S_{12}$	Li_3PO_4, Li_2SnO_3, Co_9S_8, Li_2S, Li_2SO_4
$Li_{0.5}CoO_2$, $Li_{10}SnP_2S_{12}$	Li_8SnO_6, Li_3PO_4, Li_2SnO_3, Co_9S_8, Li_2SO_4
$Li_{10}GeP_2S_{12}$, $Li_3V_2(PO_4)_3$	VS_2, $Li_4P_2O_7$, V_4GeS_8, P_4S_7, GeS_2
$Li_{10}GeP_2S_{12}$, $LiV_2(PO_4)_3$	VS_2, $Li_4P_2O_7$, $LiPO_3$, GeS_2
$Li_{10}GeP_2S_{12}$, $LiFePO_4$	FePS, FeS_2, GeS_2, $Li_4P_2O_7$, Li_3PO_4
$Li_{10}GeP_2S_{12}$, $FePO_4$	FePS, $LiPO_3$, FeS_2, GeS_2, $Li_4P_2O_7$
$Li_{10}GeP_2S_{12}$, $LiMnPO_4$	$MnPS_3$, GeS_2, MnS_2, MnP, Li_3PO_4
$Li_{10}GeP_2S_{12}$, $MnPO_4$	$Li_2MnP_2O_7$, $LiPO_3$, GeS_2, MnS_2, $Mn_2P_2O_7$
$Li_{10}GeP_2S_{12}$, $LiNi_{0.5}Mn_{1.5}O_4$	MnO, Ni_3S_4, MnS_2, Li_4GeO_4, Li_2S, Li_3PO_4
$Li_{10}GeP_2S_{12}$, $Ni_{0.5}Mn_{1.5}O_4$	$Mn_3(PO_4)_2$, MnO, Ni_3S_4, Li_2SO_4, Ni_3S_2, Mn_2GeO_4
$Li_{10}GeP_2S_{12}$, $LiMn_2O_4$	MnS_2, MnO, Li_2S, Li_3PO_4, Li_2GeO_3
MnO_2, $Li_{10}GeP_2S_{12}$	MnS_2, MnO, Li_2SO_4, Li_3PO_4, Mn_2GeO_4
$Li_{10}GeP_2S_{12}$, $LiNi_{0.8}Co_{0.15}Al_{0.05}O_2$	$LiAlO_2$, Li_2S, Li_4GeO_4, Ni_3S_2, Li_3PO_4, Co_9S_8, Li_2SO_4
$Li_{10}GeP_2S_{12}$, $Li_{0.5}Ni_{0.8}Co_{0.15}Al_{0.05}O_2$	Li_4GeO_4, Li_3AlGeO_5, Ni_3S_2, Li_3PO_4, Co_9S_8, Li_2SO_4, Ni
$Li_{10}GeP_2S_{12}$, $LiNi_{0.33}Mn_{0.33}Co_{0.33}O_2$	Co_2NiS_4, MnO, Ni_3S_4, Li_4GeO_4, MnS_2, Li_2S, Li_3PO_4
$Li_{10}GeP_2S_{12}$, $Li_{0.67}Ni_{0.33}Mn_{0.33}Co_{0.33}O_2$	Co_2NiS_4, Ni_3S_2, MnO, Li_2SO_4, Li_4GeO_4, Li_2S, Li_3PO_4
$Li_{10}GeP_2S_{12}$, $LiNiO_2$	Ni_3S_2, Li_4GeO_4, Li_2S, Li_3PO_4, Li_2SO_4
$Li_{10}GeP_2S_{12}$, NiO_2	$LiNiPO_4$, Ni_3S_2, $Ni_3(PO_4)_2$, Li_2SO_4, Ni_2GeO_4
$Li_{10}GeP_2S_{12}$, $LiCoO_2$	Li_3PO_4, Li_2S, Co_9S_8, Li_4GeO_4, Li_2SO_4
$Li_{10}GeP_2S_{12}$, $Li_{0.5}CoO_2$	Li_3PO_4, Li_2S, Co_9S_8, Li_4GeO_4, Li_2SO_4

Table S2: Predicted reaction products at cathode/SE interfaces using pseudobinary phase diagrams.

Bibliography

[1] Anubhav Jain, Geoffroy Hautier, Charles Moore, Shyue Ong, Chris Fischer, Tim Mueller, Kristin Persson, and Gerbrand Ceder. A high-throughput infrastructure for density functional theory calculations. *Comput. Mater. Sci.*, 50:2295–2310, 2011.

[2] G. Bergerhoff, R. Hundt, R. Sievers, and I David Brown. The inorganic crystal structure data base. *J. Chem. Inf. Comput. Sci.*, 23:66–69, 1983.

[3] G. Kresse and J. Furthmüller. Efficient iterative schemes for ab initio total-energy calculations using a plane-wave basis set. *Phys. Rev. B*, 54:11169–11186, 1996.

[4] G. Kresse and J. Hafner. Ab initio molecular dynamics for liquid metals. *Phys. Rev. B*, 47:558–561, 1993.

[5] G. Kresse and J. Hafner. Ab initio molecular-dynamics simulation of the liquid-metal–amorphous-semiconductor transition in germanium. *Phys. Rev. B*, 49:14251–14269, 1994.

[6] G. Kresse and J. Furthmüller. Efficiency of ab-initio total energy calculations for metals and semiconductors using a plane-wave basis set. *Comput. Mater. Sci.*, 6(1):15–50, 1996.

[7] G. Kresse and D. Joubert. From ultrasoft pseudopotentials to the projector augmented-wave method. *Phys. Rev. B*, 59:1758–1775, 1999.

[8] P. E. Blöchl. Projector augmented-wave method. *Phys. Rev. B*, 50:17953–17979, 1994.

[9] John P. Perdew, Kieron Burke, and Matthias Ernzerhof. Generalized gradient approximation made simple. *Phys. Rev. Lett.*, 77:3865–3868, 1996.

[10] Lei Wang, Thomas Maxisch, and Gerbrand Ceder. Oxidation energies of transition metal oxides within the GGA+U framework. *Phys. Rev. B*, 73:195107, 2006.

[11] Anubhav Jain, Geoffroy Hautier, Shyue Ping Ong, Charles J. Moore, Christopher C. Fischer, Kristin A. Persson, and Gerbrand Ceder. Formation enthalpies by mixing GGA and GGA+U calculations. *Phys. Rev. B*, 84:045115, 2011.

[12] Hanmei Tang, Zhi Deng, Zhuonan Lin, Zhenbin Wang, Iek-Heng Chu, Chi Chen, Zhuoying Zhu, Chen Zheng, and Shyue Ping Ong. Probing solid–solid interfacial reactions in all-solid-state sodium-ion batteries with first-principles calculations. *Chem. Mater.*, 30(1):163–173, 2018.

[13] Shyue Ping Ong, Lei Wang, Byoungwoo Kang, and Gerbrand Ceder. Li-Fe-P-O_2 phase diagram from first principles calculations. *Chem. Mater.*, 20(5):1798–1807, 2008.

[14] Yizhou Zhu, Xingfeng He, and Yifei Mo. First principles study on electrochemical and chemical stability of solid electrolyte–electrode interfaces in all-solid-state Li-ion batteries. *J. Mater. Chem. A*, 4:3253–3266, 2016.

[15] Yizhou Zhu, Xingfeng He, and Yifei Mo. Origin of outstanding stability in the lithium solid electrolyte materials: Insights from thermodynamic analyses based on first-principles calculations. *ACS Appl. Mater. Interfaces*, 7(42):23685–23693, 2015.

[16] William D. Richards, Lincoln J. Miara, Yan Wang, Jae Chul Kim, and Gerbrand Ceder. Interface stability in solid-state batteries. *Chem. Mater.*, 28(1):266–273, 2016.

[17] Lincoln J. Miara, William Davidson Richards, Yan E. Wang, and Gerbrand Ceder. First-principles studies on cation dopants and electrolyte|cathode interphases for lithium garnets. *Chem. Mater.*, 27(11):4040–4047, 2015.

[18] Manas Likhit Holekevi Chandrappa, Ji Qi, Chi Chen, Swastika Banerjee, and Shyue Ping Ong. Thermodynamics and kinetics of the cathode–electrolyte interface in all-solid-state Li–S batteries. *J. Am. Chem. Soc.*, 144(39):18009–18022, 2022.

[19] Shyue Ong, William Richards, Anubhav Jain, Geoffroy Hautier, Michael Kocher, Shreyas Cholia, Dan Gunter, Vincent Chevrier, Kristin Persson, and Gerbrand Ceder. Python materials genomics (pymatgen): A robust, open-source python library for materials analysis. *Comput. Mater. Sci.*, 68:314–319, 2013.

[20] Robert J. Friauf. Correlation effects for diffusion in ionic crystals. *J. Appl. Phys.*, 33(1):494–505, 1962.

[21] J Ibarra, Alejandro Varez, Carlos Leon, J. Santamaría, Leticia Torres-Martínez, and Jesús Sanz. Influence of composition on the structure and conductivity of the fast ionic conductors $La_{2/3x}Li_{3x}TiO_3$ ($0.03 \leq x \leq 0.167$). *Solid State Ion.*, 134:219–228, 2000.

[22] Stefan Zahn, Jürgen Janek, and Doreen Mollenhauer. A simple ansatz to predict the structure of $Li_4Ti_5O_{12}$. *J. Electrochem. Soc.*, 164(2):A221, 2016.

[23] Xingyu Qu, Zhenlu Yu, Dingshan Ruan, Aichun Dou, Mingru Su, Yu Zhou, Yunjian Liu, and Dewei Chu. Enhanced electrochemical performance of ni-rich cathode materials with $Li_{1.3}Al_{0.3}Ti_{1.7}(PO_4)_3$ coating. *ACS Sustain. Chem. Eng.*, 8(15):5819–5830, 2020.

[24] Yue Deng, Christopher Eames, Jean-Noël Chotard, Fabien Lalère, Vincent Seznec, Steffen Emge, Oliver Pecher, Clare P. Grey, Christian Masquelier, and M. Saiful Islam. Structural and mechanistic insights into fast lithium-ion conduction in Li_4SiO_4-Li_3PO_4 solid electrolytes. *J. Am. Chem. Soc.*, 137(28):9136–9145, 2015.
